# Probiotics for the Prevention of Ventilator-Associated Pneumonia: An Updated Systematic Review and Meta-Analysis of Randomised Controlled Trials

**DOI:** 10.3390/nu14081600

**Published:** 2022-04-12

**Authors:** Huzaifa Ahmad Cheema, Abia Shahid, Muhammad Ayyan, Biah Mustafa, Afra Zahid, Maurish Fatima, Muhammad Ehsan, Farwa Athar, Natalie Duric, Tamas Szakmany

**Affiliations:** 1 Department of Medicine, King Edward Medical University, Lahore 54000, Pakistan; abiashahid126@gmail.com (A.S.); iamayyan77@gmail.com (M.A.); biahmustafa97@gmail.com (B.M.); afrazahid1234@gmail.com (A.Z.); maurishfatima16@gmail.com (M.F.); m.ehsanqadri@gmail.com (M.E.); farwaathar61999@gmail.com (F.A.); 2 Critical Care Directorate, The Grange University Hospital, Aneurin Bevan University Health Board, Cwmbran NP44 2XJ, UK; natalie.duric@gmail.com; 3 Department of Anaesthesia, Intensive Care and Pain Medicine, Division of Population Medicine, Cardiff University, Cardiff CF14 4XN, UK

**Keywords:** meta-analysis, probiotics, synbiotics, ventilator-associated pneumonia, VAP

## Abstract

Background: Presently, there is conflicting evidence regarding the efficacy of probiotics in the prevention of ventilator-associated pneumonia (VAP). This meta-analysis was conducted to update current clinical evidence and evaluate the efficacy and safety of probiotics for the prevention of VAP. Methods: We searched three databases and two trial registers to retrieve randomised controlled trials (RCTs) comparing probiotics or synbiotics with placebo or standard treatment for the prevention of VAP in adult patients receiving mechanical ventilation in the intensive care unit (ICU). Results: Our meta-analysis included 18 RCTs involving 4893 patients. Our results showed that probiotics may reduce the incidence of VAP (RR 0.68, 95% CI: 0.55–0.84; low certainty). However, in our subgroup and sensitivity analyses, the effect was not significant in double-blind studies, and in studies with a low risk of bias in the randomisation process. Probiotics reduced the length of ICU stay (MD −2.22 days, 95% CI: −4.17 to −0.28; moderate certainty) and the duration of antibiotic use (MD −1.25 days, 95% CI −1.86 to −0.64; moderate certainty). Conclusions: Probiotics may reduce the incidence of VAP but due to the low quality of pooled evidence, the use of probiotics warrants caution. Further, large-scale, high-quality RCTs need to be conducted to provide conclusive evidence.

## 1. Introduction

Ventilator-associated pneumonia (VAP) is defined as a pulmonary infection in patients who have been mechanically ventilated continuously for at least a forty-eight-hour period [1]. It is one of the most frequent nosocomial infections in patients requiring mechanical ventilation. Studies have reported that VAP affects between 5–40% of patients requiring mechanical ventilation depending on the country, intensive care unit (ICU) type and diagnostic criteria for VAP [2,3]. VAP is associated with a high rate of all-cause mortality and a prolonged duration of mechanical ventilation and ICU stay [1].

There have been previous attempts to manipulate the microbiome using pharmacological strategies to reduce the incidence of VAP including the use of antibiotics for selective digestive decontamination (SDD) or selective oral decontamination (SOD). Neither of these has conclusively shown to be beneficial and current guidance, which was updated five years ago, is equivocal in recommending either strategy [4]. Another strategy to try to address the imbalance in the microbiome of the critically ill patient is the use of probiotics or synbiotics. Probiotics are commercially available living microbial agents and synbiotics are a combination of probiotics and prebiotics, both of which have proposed beneficial effects on the health of the host [5].

It has been suggested that the preventive effect of probiotics in VAP is achieved through maintenance of aerodigestive microbial equilibrium and modulation of immune function both locally and systematically. Reduction in the growth of potentially pathogenic microbes, bacterial translocation, toll-like receptor-mediated upregulation of immune response and potentiating the function of the gut-mucosal barrier are the proposed mechanisms of action [5,6,7,8]. Probiotics reportedly have minimal contraindications to their use [5]. Their ability to inhibit the colonisation of the digestive tract with antibiotic-resistant bacteria may confer some advantage over the use of antibiotics in critically unwell patients in the ICU [9].

Several trials have shown beneficial effects of probiotic therapy in the prevention of VAP in the critically ill [10,11]. Subsequent meta-analyses have been conducted to evaluate its efficacy in the prevention of VAP [12,13,14]. These meta-analyses, however, were based on trials with small sample sizes limiting their wider applicability. Moreover, safety outcomes were not assessed. Recently, the results of a large, multicenter RCT, Probiotics to prevent Severe Pneumonia and Endotracheal Colonisation Trial (PROSPECT), were published which did not find a reduced incidence of VAP with the use of probiotics [15].

In this meta-analysis, including the recently published PROSPECT trial’s results, we aimed to comprehensively update the clinical evidence to resolve the inconsistencies in the existing literature and determine the efficacy and safety of the use of probiotics or synbiotics in the prevention of VAP in critically ill adults.

## 2. Materials and Methods

Our meta-analysis was conducted according to the guidance presented in the *Cochrane Handbook for Systematic Reviews of Interventions* and reported according to the Preferred Reporting Items for Systematic Reviews and Meta-Analysis (PRISMA) statement (Supplementary Table S1) [16,17]. This study did not require ethical approval. Our protocol is registered with PROSPERO, The International Prospective Register of Systematic Reviews (CRD42021285401).

### 2.1. Data Sources and Searches

We searched the following online databases and trial registers from inception to January 2021: Cochrane Central Register of Controlled Trials (CENTRAL, via The Cochrane Library), MEDLINE (via Ovid), Embase (via Ovid), ClinicalTrials.gov and WHO International Clinical Trials Registry Platform (ICTRP) portal. Grey literature was identified through a search of Open Access Theses and Dissertations (OATD) and ProQuest Dissertations and Theses Global (PQDT). We also screened reference lists of included studies and similar systematic reviews to identify studies related to our topic. Additionally, we undertook forward citation tracking using the Web of Science to identify further eligible articles citing any of the included studies and relevant systematic reviews.

The complete search strategy for each database is given in Supplementary Table S2.

### 2.2. Eligibility Criteria

The inclusion criteria were as follows: (1) study design: randomised controlled trial; (2) patient population: Adult (>18 years of age) critically ill patients who received mechanical ventilation; (3) intervention: probiotics or synbiotics compared to placebo or usual care and (4) outcome: incidence of VAP.

The exclusion criteria were as follows: (1) All study designs other than RCTs, such as quasi-randomised trials and observational studies; (2) studies conducted on animals or minors and (3) studies comparing a type of probiotic with another type of probiotic or synbiotic.

### 2.3. Study Selection and Data Abstraction

We imported all the studies obtained through our online searching into Mendeley Desktop 1.19.8 (Mendeley Ltd., Amsterdam, The Netherlands) and removed the duplicate articles. Two authors (A.S and M.F) independently carried out title and abstract screening and excluded irrelevant articles. We then performed full-text screening on the remaining studies and finalised the studies conforming to our eligibility criteria. Any disagreements over the selection of studies were settled by a third author (H.A.C). We presented the selection process in the form of a PRISMA flow chart.

Two review authors (M.A and A.Z) extracted data from the included studies into an Excel spreadsheet using a pre-piloted data extraction form. Extracted data were compared and any discrepancies were resolved through discussion with a third reviewer (M.E). We extracted information on study characteristics (including authors, date of publication, study design, type of trial and follow-up duration), populations (including age and gender), interventions (including type, dose, duration and mode of intervention), comparators and outcomes (primary and additional outcomes). 

### 2.4. Outcome Measures

The primary outcome measure was the incidence of VAP. The secondary outcome measures were duration of mechanical ventilation, length of ICU stay, length of hospital stay, ICU mortality, hospital mortality, 28/30-day mortality, duration of antibiotic use, the incidence of diarrhoea, adverse events and serious adverse events of probiotic intervention.

### 2.5. Risk of Bias Assessment

We assessed the risk of bias in the included studies using the revised Cochrane Risk of Bias Tool for randomised controlled trials (RoB 2.0) [18]. RoB 2.0 addresses five domains: (1) bias arising from the randomisation process; (2) bias due to deviations from intended interventions; (3) bias due to missing outcome data; (4) bias in the measurement of the outcome and (5) bias in the selection of the reported result. Two authors (F.A and M.E) independently assessed the risk of bias for each included study as low, high, or some concerns of bias. Any disagreements in the evaluation of the risk of bias were resolved by discussion to reach a consensus between the two authors, with a third author (M.A) acting as an arbiter if necessary.

### 2.6. Data Synthesis

For each trial, we reported dichotomous outcomes as relative risk (RR) along with 95% confidence intervals. We converted medians and IQRs to means and SDs for uniform analyses using the methods described by Wan and colleagues [19]. We reported continuous outcomes as mean difference (MD) along with 95% confidence intervals. Statistical analysis was performed using Review Manager (RevMan, Version 5.4; The Cochrane Collaboration, Copenhagen, Denmark). We used DerSimonian and Laird random-effects model to perform meta-analyses. For each synthesis, we calculated the Chi^2^ test and I^2^ statistic to detect the presence of heterogeneity and quantify it, respectively. We interpreted I^2^ values according to the *Cochrane Handbook for Systematic Reviews of Interventions*, [16]. *p* < 0.10 was considered statistically significant for the Chi^2^ test [16]. The safety outcomes for which meta-analyses could not be conducted were analysed in a narrative form. We also presented the study characteristics and findings of the included studies in the form of tables.

For outcomes where the number of studies was at least 10, we constructed a funnel plot and ran the Egger’s test to check funnel plot asymmetry using Jamovi (version 1.8) MAJOR module which is based on the metafor package for R [20]. Publication bias is present when the *p*-value is less than 0.10. For outcomes where there were less than 10 studies, we constructed Doi plots and calculated Luis Furuya-Kanamori (LFK) index using MetaXL version 5.3 (EpiGear International Pty, Sunrise Beach, QLD, Australia) to evaluate publication bias. The LFK index has greater sensitivity and power than the Egger test and hence, is suitable for a lower number of studies [21].

### 2.7. Subgroup and Sensitivity Analyses

To explore the causes of heterogeneity, we performed subgroup analyses on the basis of trial design (double-blind, single-blind, and open-label), type of intervention (probiotic and synbiotic), type of comparator (placebo and others), and definition of VAP (clinically diagnosed, microbiologically confirmed, and inexplicable diagnostic criteria). The subgroup analyses were conducted on the primary outcome and secondary outcomes with substantial heterogeneity. *p* < 0.10 was considered to be statistically significant for the test for subgroup differences [22].

For the primary outcome, we also conducted a sensitivity analysis by excluding trials with some concerns of bias in the randomisation process.

### 2.8. Certainty of Evidence Assessment

Two authors (A.S and H.A.C) independently assessed the certainty of the evidence. We used the five Grades of Recommendation, Assessment, Development, and Evaluation (GRADE) considerations (study limitations, consistency of effect, imprecision, indirectness, and publication bias) to assess the certainty of the body of evidence [23,24]. We considered pooled effects imprecise if the optimal information size criterion was not met, or the associated 95% CIs included the null effect and appreciable benefit or harm [25]. We judged quality as high (further research is very unlikely to change our confidence in the estimate of effect), moderate (further research is likely to have an important impact on our confidence in the estimate of effect and may change the estimate), low (further research is very likely to have an important impact on our confidence in the estimate of effect and is likely to change the estimate), or very low (very uncertain about the estimate of effect). We presented our findings in the form of a ‘summary of findings’ table.

## 3. Results

**Figure 1:**
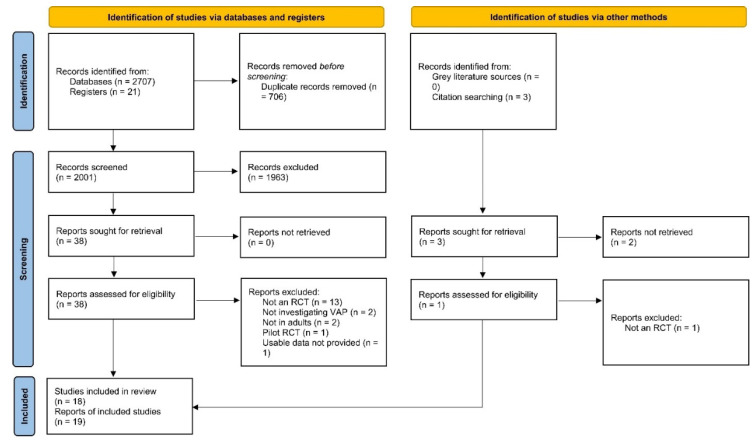
PRISMA 2020 flow chart. Flow chart of included and excluded trials. PRISMA, Preferred Reporting Items for Systematic Reviews and Meta-Analyses.

The detailed screening process is depicted in Figure 1. After deduplication and screening, a total of 19 reports were included in our review [10,11,15,26,27,28,29,30,31,32,33,34,35,36,37,38,39,40,41]. These 19 reports provided data on 18 RCTs comprising a total of 4893 participants (2409 in the probiotic/synbiotic group and 2484 in the control group) from 13 countries. Probiotics (12 RCTs) or synbiotics (6 RCTs) were compared to placebo or control groups. VAP was diagnosed clinically in seven studies and microbiologically in eight studies whereas three studies had an inexplicit diagnostic criterion. The detailed characteristics of included studies are shown in Table 1.

### 3.1. Risk of Bias in Included Studies

**Figure 2:**
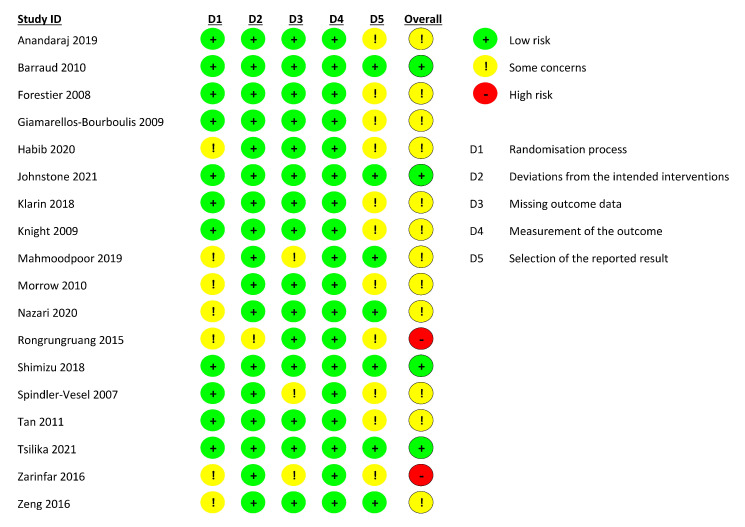
Risk of bias assessment for each included study.

Overall, only four studies (22.2%) were judged to be at low risk of bias while two studies (11.1%) were at a high risk of bias due to some concerns of bias in multiple domains (Figure 2). The remaining studies (66.7%) were found to be at some risk of bias, mostly in the domains of randomisation process and selection of reported results. The most common flaw was a lack of data about allocation concealment and no availability of a trial registry record containing an analysis plan.

### 3.2. Effects of Interventions

#### Primary Outcome: Incidence of VAP

**Figure 3:**
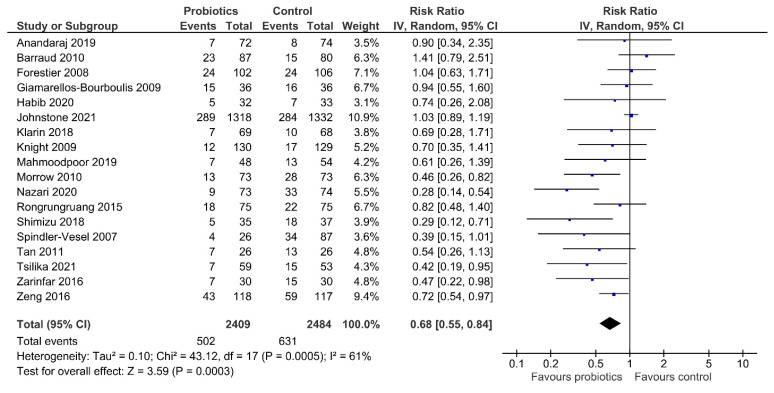
Comparison of incidence of VAP between patients receiving probiotics/synbiotics or control. VAP, ventilator-associated pneumonia; IV, inverse variance.

Our meta-analysis indicated that treatment with probiotics or synbiotics significantly reduced the incidence of VAP compared with the control group (RR 0.68, 95% CI 0.55–0.84; Figure 3). The estimated heterogeneity was substantial (I^2^ = 61%). The number needed to treat (NNT) to prevent one incidence of VAP in mechanically ventilated patients was 11. Egger’s test showed evidence of funnel plot asymmetry (*p* = 0.012; Supplementary Figure S1). The overall quality of evidence was rated as low due to concerns about inconsistency and publication bias (Table 2).

## 4. Secondary Outcomes

### 4.1. Duration of Mechanical Ventilation

There was no significant difference in the duration of mechanical ventilation between the intervention arm and the control group (MD −1.22 days, 95% CI −3.25–0.81; Supplementary Figure S2). The interstudy heterogeneity was considerable (I^2^ = 96%). The funnel plot showed no evidence of asymmetry (Egger’s *p*-value of 0.678; Supplementary Figure S3). The quality of evidence was downgraded to low due to inconsistency and imprecision (Table 2).

### 4.2. Length of ICU Stay

The use of probiotics or synbiotics was associated with a statistically significant reduction in the length of ICU stay (MD −2.22 days, 95% CI −4.17 to −0.28; Supplementary Figure S4). The statistical heterogeneity between studies was considerable (I^2^ = 96%). No funnel plot asymmetry was detected by Egger’s test (*p* = 0.107; Supplementary Figure S5). The quality of evidence was judged to be moderate with concerns about inconsistency only (Table 2).

### 4.3. Length of Hospital Stay

There was no significant difference in the length of hospital stay between the two groups (MD −1.47 days, 95% CI −4.06–1.12) with a substantial to a considerable amount of heterogeneity (I^2^ = 77%; Supplementary Figure S6). The Doi plot returned major asymmetry (LFK index = −3.29; Supplementary Figure S7). The certainty of the evidence was downgraded to very low due to concerns of inconsistency, imprecision and publication bias (Table 2).

### 4.4. ICU, Hospital and 28/30-Day Mortality

There was no significant difference between the patients treated with various probiotics or synbiotics and the control group for ICU, hospital and 28/30-day mortality (RR 0.96, 95% CI 0.85–1.09; RR 0.94, 95% CI 0.84–1.05 and RR 0.94, 95% CI 0.66–1.32, respectively; Supplementary Figure S8). Estimated heterogeneity was low for all three outcomes (I^2^ = 0%). Doi plots indicated borderline minor asymmetry for ICU mortality (LFK index = 1.07; Supplementary Figure S9) and major asymmetry for hospital and 28/30-day mortality (LFK index = −4.74 and −2.00, respectively; Supplementary Figures S10 and S11). The quality of evidence was rated as moderate for ICU mortality due to imprecision while for hospital and 28/30-day mortality it was low due to imprecision and suspected publication bias (Table 2).

### 4.5. Duration of Antibiotic Use

Our meta-analysis indicated a shorter duration of antibiotic use in the intervention arm than in the control group (MD −1.25 days, 95% CI −1.86 to −0.64) with low heterogeneity (I^2^ = 0%; Supplementary Figure S12). The Doi plot suggested major asymmetry (LFK index = −7.42; Supplementary Figure S13). The credibility of evidence was judged to be moderate due to concerns about publication bias (Table 2).

### 4.6. Incidence of Diarrhoea

There was no significant difference in the incidence of diarrhoea between the intervention and control arms (RR 0.98, 95% CI 0.86–1.11; Supplementary Figure S14). Estimated heterogeneity was low (I^2^ = 19%). Upon inspection of the Doi plot, we found major asymmetry (LFK index = −6.39; Supplementary Figure S15) which raised suspicion of publication bias; hence, the quality of evidence was downgraded to moderate (Table 2).

### 4.7. Any Adverse Effects of the Probiotics or Synbiotics

Only two studies reported any adverse effects of treatment [15,31]. Johnstone and colleagues reported that the risk of adverse events was significantly higher in the probiotic group (15/1318) than in the placebo group (1/1332) with an OR of 14.02 (95% CI 1.79–109.58) [15]. Out of 15 events in the probiotic arm, two were SAEs defined as “Lactobacillus isolates that resulted in persistent or significant disability or incapacity, were life-threatening, or resulted in death.” Tsilika and colleagues reported no significant difference in the incidence of adverse events between the two groups (*p* = 0.328) with 52 (88.1%) and 50 (94.3%) patients experiencing at least one SAE in the probiotic and the placebo groups, respectively but did not provide a definition for this outcome [31].

### 4.8. Subgroup Analysis

Two of our *a priori* defined subgroup analyses (according to the route of administration of probiotics and pathogen implicated in VAP) were not carried out due to large variation among the studies resulting in not enough studies in each subgroup. Instead, we decided to carry out a subgroup analysis based on the type of intervention used (probiotic or synbiotic).

#### 4.8.1. Blinding

There was no significant difference in the incidence of VAP between the treatment and control arms in the double-blind studies subgroup (RR 0.80, 95% CI 0.63–1.01; 11 studies; Table 3). The test for subgroup differences was significant (*p* = 0.002). For the secondary outcomes with high heterogeneity (duration of mechanical ventilation, length of ICU stay and length of hospital stay), there was no significant difference between the subgroups (Table 3).

#### 4.8.2. Type of Intervention

Synbiotics were associated with a greater benefit in reducing the incidence of VAP (RR 0.50, 95% CI 0.32–0.79; 6 studies) compared with probiotics (RR 0.77, 95% CI 0.63–0.96; 12 studies) with the test for subgroup differences returning a significant *p*-value (0.09). There were no significant between-subgroup differences in the secondary outcomes (Table 3).

#### 4.8.3. Type of Comparator

There was no significant difference between the subgroups of placebo and other comparators in the incidence of VAP (*p* = 0.55) or the secondary outcomes (Table 3).

#### 4.8.4. Diagnostic Criteria for VAP

There was no significant difference in the incidence of VAP between the studies that diagnosed VAP clinically, those that confirmed VAP microbiologically and those with inexplicit diagnostic criteria (*p* = 0.20; Table 3).

### 4.9. Sensitivity Analysis

After excluding trials at some concerns of bias in the randomisation process, the incidence of VAP did not differ significantly between the intervention and control arms (RR 0.78, 95% CI 0.60–1.00; I^2^ = 52%; Supplementary Figure S16).

## 5. Discussion

This meta-analysis of 18 RCTs involving 4893 patients found a decrease in the incidence of VAP with the use of either probiotics or synbiotic therapy. The quality of evidence is low, however, owing to inconsistency between RCTs and suspicion of publication bias. Furthermore, the use of probiotic or synbiotic therapy was associated with a decrease in the duration of ICU stay and duration of antibiotic use (moderate-quality evidence). The use of probiotics, however, did not significantly change the duration of mechanical ventilation; the length of hospital stay; ICU, hospital or 28/30 day mortality, or the incidence of diarrhoea. The quality of evidence for these secondary outcomes was low to moderate. Data regarding the safety of probiotics come mainly from the PROSPECT trial, indicating that probiotics may increase the risk of adverse effects [15].

The results of our meta-analysis are in accordance with the outcomes of the recent meta-analyses which also found a significant reduction in the incidence of VAP in the probiotic group [12,13,14,42,43]. Our meta-analysis takes into consideration PROSPECT, the largest RCT conducted to date with a total of 2650 patients [15]. Importantly, this trial concluded that the administration of a probiotic does not reduce the incidence of VAP in critically ill patients contrary to the findings of several preceding smaller trials. Clinical trials conducted on a smaller scale in critical care settings inadvertently tend to inflate their findings and over-report the true relation between the intervention and the outcome [44]. Although recent meta-analyses established that the administration of probiotics may decrease the incidence of VAP, this was in disagreement with the findings of PROSPECT. It is important to note that meta-analyses of smaller trials are more susceptible to biases, especially publication bias. Consequently, they often report positive findings not substantiated by subsequent larger RCTs [45].

The pathogenesis of VAP is thought to involve bacterial colonisation of the upper airways and translocation of contaminated secretions beyond the cuff of the endotracheal tube due to microaspirations [1]. Changing the microflora of the upper respiratory tract using probiotics and reducing the colonisation by pathogenic species has been an attractive option to prevent the development of VAP [1]. The reduction of bacterial translocation, improvement of local host defences and modulation of local and general immunity against more virulent species of bacteria have been shown to be associated with probiotic treatment [5,6].

In our meta-analysis, although probiotics were found to be beneficial in the reduction of VAP, the overall quality of evidence was low substantially reducing our confidence in the pooled estimate. Our inferences are further weakened by the results of our subgroup and sensitivity analyses which indicate that in studies with double-blinding and studies at low risk of bias in the randomisation process, probiotics do not decrease the incidence of VAP. Bias due to inadequate allocation concealment in RCTs is associated with exaggerated intervention effects which may have been the factor that influenced our results [46].

The quality of evidence using the recommended GRADE ranking was only assessed by two previous studies [12,42]. Bo and colleagues reported it to be low/similar to our rating, whereas Ji and colleagues judged their aggregated evidence to be of high quality by upgrading it due to a large estimated effect [12,42]. However, as suggested by the *Cochrane Handbook for Systematic Reviews of Interventions*, the effect estimate should be at least RR < 0.5 to consider upgrading it [16]. Therefore, we feel that Ji and others were incorrect to upgrade the quality of evidence, as the summary RR calculated both by them and our meta-analysis was >0.5 [12].

Additionally, our meta-analysis showed that synbiotics were associated with a greater benefit in reducing the incidence of VAP as compared to probiotics. This should be interpreted with caution as subgroup comparisons are observational in nature and may be confounded by other study-level characteristics. Nevertheless, in a recent network meta-analysis, synbiotics were found to be more effective than probiotics for the prevention of nosocomial infections in critically ill adults, corroborating our findings [47]. 

It is important to note that other general strategies which aim at reducing the time spent on mechanical ventilation, such as the implementation of the ABCDEF bundle, promoting light sedation and early spontaneous breathing trials to assess readiness for extubation, might produce similar or even superior results [48].

## 6. Strengths and Limitations

The main strength of our study is the broad inclusion criteria, which enabled us to include 18 RCTs in the adult population, resulting in the largest contemporary meta-analysis to date. Our meta-analysis extends the results of previous ones by taking a significantly larger sample size into consideration, enabling a better investigation of heterogeneity and a more accurate evaluation of the certainty of evidence. We used the certainty of the evidence for each outcome to inform our conclusions. This not only helped gauge the importance of the findings of our meta-analysis but also sets it apart from the pre-existing work. The inclusion of studies from diverse settings, populations and countries and an assessment of the safety of probiotic/synbiotic therapy add to the strengths of this study.

There are a few limitations, however, to the current meta-analysis. Firstly, the majority of studies (66.7%) were found to be at some concern of bias while two studies were at a high risk of bias. Heterogeneity resulting from differences in the mean age of participants across the pool of studies, differences in the probiotic employed as well as their dosages and routes of administration further limits our findings. The definition of VAP and the diseased states also varied among the included studies and none of the studies evaluated the use of VAP in immunocompromised individuals, thereby excluding an important population. It has been shown previously that the different definitions of VAP can substantially change the incidence reported, hence our positive finding should be interpreted with extreme caution [49,50,51]. Finally, our meta-analysis is based on aggregate-level data as we did not have access to patient-level data.

## 7. Implications for Practice and Research

Based on the data of 4893 patients and adherence to a strict inclusion criterion, our meta-analysis suggested that the administration of probiotics or synbiotics in critically ill adult patients may decrease the incidence of VAP. However, inconsistency between patient and intervention characteristics, study limitations and an increased risk of adverse effects caution their use as a standard prophylaxis strategy. Moreover, the lack of data on various formulations of probiotics and their mode of action in different diseased states warrants careful usage.

In the future, large, multicenter RCTs should be conducted that study the effect of probiotics on VAP incidence, ensuring VAP diagnosis and incidence are reported utilising a consensus VAP definition and externally validated diagnostic criteria. The ability to further analyse pre-defined patient subpopulations may be of particular interest, e.g., trauma patients where previously the greatest benefit of probiotic therapy has been observed [47]. Trials should be focused on those probiotic or synbiotic formulations that have been associated with beneficial effects and should ensure they are sufficiently powered to detect adverse effects too. Concurrently, studies investigating the mechanisms of action of different probiotic species should be conducted since it is simplistic to assume a generalised view of the mechanisms of action of different strains.

## 8. Conclusions

The results of our meta-analysis indicate that the administration of probiotics may be useful in reducing the incidence of VAP in critically unwell adults. However, since this benefit was not observed in analyses restricted to double-blinded or adequately randomised studies, the importance of this finding becomes questionable. There was also a significant increase in the risk of adverse effects with probiotic use. Further large-scale, well-designed, randomised, multicenter trials are required to validate the current findings and determine the effectiveness of different strains of probiotics.

## Figures and Tables

**Table 1:** Characteristics of included RCTs.

Study ID (First Author, Year)	Country of Origin	Trial Design	Study Follow-Up Duration	No. of Patients (Total [Probiotic Group vs. Control]) *	Age (Years) *†	Male (%) *	Disease Types	Method of Administration	Experimental Intervention	Comparator Intervention	Diagnostic Criteria for VAP
Spindler-Vesel and colleagues, 2007 [36]	Solvenia	SC, SB	ICU stay	113 (26 vs. 87)	45.8 ± 22.9 vs. 37.1 ± 36.4	77.9 (total)	Multi trauma	Intragastric tube	Synbiotic 2000 FORTE	Group A: glutamine; Group B: fermentable fibres; Group C: peptide diet	Inexplicable
Forestier and colleagues, 2008 [41]	France	SC, DB	ICU stay	208 (102 vs. 106)	60 (18–91) vs. 57 (18–80)	63.7 vs. 76.4	Multidisease and trauma	Nasogastric tube/oral	*L. casei rhamnosus* (10^9^), bid	Placebo (growth medium without bacteria), bid	Microbiologically confirmed
Giamarellos-Bourboulis and colleagues, 2009 [28,29]	Greece	MC, DB	28 days	72 (36 vs. 36)	52.9 ± 19.0 vs. 55.9 ± 18.0	NA	Multi trauma	Nasogastric tube/gastrostomy	Synbiotic 2000 FORTE, qd	Placebo	Clinically diagnosed
Knight and colleagues, 2009 [32]	UK	SC, DB	Hospital stay	259 (130 vs. 129)	49.5 ± 19.6 vs. 50.0 ± 18.5	62.3 vs. 62.0	Multi-disease	Nasogastric/orogastric tube	Synbiotic 2000 FORTE, bid	Crystalline cellulose-based placebo	Microbiologically confirmed
Barraud and colleagues, 2010 [27]	France	SC, DB	90 days	167 (87 vs. 80)	59.1 ± 15.9 vs. 61.8 ± 15.5	86.8 vs. 79.5	Multi-disease	Enteral feeding tube	Ergyphilus capsules	Placebo	Microbiologically confirmed
Morrow and colleagues, 2010 [10]	Nebraska	SC, DB	25 days	138 (68 vs. 70)	52.5 ± 19.3 vs. 54.6 ± 16.3	58.9 vs. 58.9	Multi-disease and Trauma	Slurry to oropharynx/Nasogastric tube	*Lactobacillus rhamnosus GG*, bid	Plant starch inulin	Microbiologically confirmed
Tan and colleagues, 2011 [37]	China	SC, SB	21 days	52 (26 vs. 26)	40.5 ± 13.0 vs. 40.8 ± 12.8	73.1 vs. 80.8	Severe traumatic brain-injured	nasogastric tube/oral	Golden bifid 7 sachets, tid	Placebo	Microbiologically confirmed
Rongrungruang and colleagues, 2015 [34]	Thailand	SC, OP	90 days	150 (75 vs. 75)	73.1 ± 13.2 vs. 69.0 ± 18.5	40.0 vs. 42.7	Multi-disease	Oral care and enteral feeding tube	*Lactobacillus*, 80 mL, oral care, qd, 80 mL, enteral feeding, qd	Placebo	Clinically diagnosed
Zarinfar and colleagues, 2016 [39]	Iran	SC, DB	NA	60 (30 vs. 30)	41.4 ± 18.8 vs. 48.2 ± 18.9	70.0 vs. 66.7	Multi-disease	Slurry to oropharynx	*Lactobacillus rhamnosus GG*, 3 × 10^9^ CFU, tid	Placebo	Clinically diagnosed
Zeng and colleagues, 2016 [40]	China	MC, OP	14 days	235 (118 vs. 117)	50.2 ± 18.2 vs. 54.6 ± 17.9	61.9 vs. 55.6	Multi-disease	nasogastric tube	Probiotics capsule containing live *Bacillus subtilis* and *Enterococcus faecalis* (Medilac-S) 0.5 g three times daily plus standard preventive strategies	Standard preventive strategies	Microbiologically confirmed
Klarin and colleagues, 2018 [31]	Sweden	MC, OP	180 days	137 (69 vs. 68)	66 (57–76) vs. 65.5 (53.75–75)	58.0 vs. 52.9	Multi-Disease	Oral care	*Lactobacillus plantarum 299*, 10^10^ CFU, bid	standard 0.1% CHX solution plus toothpaste	Clinically diagnosed
Shimizu and colleagues, 2018 [35]	Japan	SC, SB	28 days	72 (35 vs. 37)	74 (64–82) vs. 74 (64–81)	71.4 vs. 59.5	Sepsis	Nasogastric tube/orally	Synbiotics: Seichoyaku, 3 g and galactooligosaccharides 10 g, qd	No treatment	Inexplicable
Anandaraj and colleagues, 2019 [26]	India	SC, DB	ICU stay	146 (72 vs. 74)	42.0 ± 17.0 vs. 43.0 ± 17.0	60.0 vs. 57.0	Multi-disease	Oral slurry/nasogastric	*Lactobacillus* 2 × 10^9^ CFU, bid	Inert Powder	Clinically diagnosed
Mahmoodpoor and colleagues, 2019 [33]	Iran	MC, DB	14 days	100 (48 vs. 52)	59.1 ± 12.9 vs. 57.5 ± 14.5	54.2 vs. 53.7	Multi-disease	Nasogastric tube	LactoCare capsule (synbiotic), bid	Placebo (sterile maize starch)	Microbiologically confirmed
Habib and colleagues, 2020 [30]	Egypt	SC, DB	ICU stay	65 (32 vs. 33)	39.5 ± 7.7 (total)	80.0 (total)	Multi trauma	Orogastric/nasogastric tube	Lacteol Forte Sachet, tid	Placebo sachet	Inexplicable
Nazari and colleagues, 2020 [11]	Iran	MC, SB	ICU stay	147 (73 vs. 74)	52.2 ± 4.1 vs. 53.0 ± 4.0	67.1 vs. 70.3	Multi-trauma	Nasogastric tube	LactoCare capsule (synbiotic), bid	Starch	Clinically diagnosed
Johnstone and colleagues, 2021 [15]	Canada, USA, Saudi Arabia	MC, DB	Hospital stay	2650 (1318 vs. 1332)	60.1 ± 16.2 vs. 59.6 ± 16.8	59.0 vs. 60.8	Multi-disease	Nasogastric/orogastric/nasoduodenal/oroduodenal tube	*Lactobacillus rhamnosus GG*, 10^10^ CFU, bid	Microcrystalline cellulose	Clinically diagnosed
Tsilika and colleagues, 2021 [38]	Greece	MC, DB	30 days	112 (59 vs. 53)	38.1 ± 17.2 vs. 43.8 ± 14.4	91.5 vs. 75.5	Multi-trauma	Nasogastric/gastrostomy tube	LactoLevure (a four-probiotic preparation), bid	Powdered glucose polymer	Microbiologically confirmed

VAP, ventilator-associated pneumonia; CFU, colony-forming units; ICU, intensive care unit; CHX, chlorhexidine; SC, single-centre; MC, multi-centre; OP, open-label; SB, single-blind; DB, double-blind; NA, not available. * Data express the comparison of results between the probiotic group and control group. † Data is expressed as mean ± standard deviation (SD) or median (IQR).

**Table 2:** Grading of recommendations assessment, development, and evaluation (GRADE) summary of findings.

Outcome	No. of Participants (Studies)	Effect Estimate (95% CI)	Risk Of Bias	Inconsistency	Indirectness	Imprecision	Publication Bias	Quality of Evidence (GRADE)
VAP incidence	4893 (18)	RR 0.68 (0.55–0.84)	Not serious	Serious	Not serious	Not serious	Suspected	⊕⊕⊖⊖ LOW
Duration of mechanical ventilation (days)	4182 (12)	MD −1.22 (−3.25–0.81)	Not serious	Serious	Not serious	Serious	Undetected	⊕⊕⊖⊖ LOW
Length of ICU stay (days)	4493 (15)	MD −2.22 (−4.17 to −0.28)	Not serious	Serious	Not serious	Not serious	Undetected	⊕⊕⊕⊖ MODERATE
Length of hospital stay (days)	3907 (9)	MD −1.47 (−4.06–1.12)	Not serious	Serious	Not serious	Serious	Suspected	⊕⊖⊖⊖ VERY LOW
ICU mortality	3872 (9)	RR 0.96 (0.85–1.09)	Not serious	Not serious	Not serious	Serious	Undetected	⊕⊕⊕⊖ MODERATE
Hospital mortality	3673 (8)	RR 0.94 (0.84–1.05)	Not serious	Not serious	Not serious	Serious	Suspected	⊕⊕⊖⊖ LOW
28/30-day mortality	553 (5)	RR 0.94 (0.66–1.32)	Not serious	Not serious	Not serious	Serious	Suspected	⊕⊕⊖⊖ LOW
Duration of antibiotic use (days)	497 (4)	MD −1.25 (−1.86 to −0.64)	Not serious	Not serious	Not serious	Not serious	Suspected	⊕⊕⊕⊖ MODERATE
Incidence of diarrhea	3710 (9)	RR 0.98 (0.86–1.11)	Not serious	Not serious	Not serious	Not serious	Suspected	⊕⊕⊕⊖ MODERATE

CI, confidence interval; MD, mean difference; RR, risk ratio.

**Table 3:** Subgroup analysis.

Subgroup		Incidence of VAP		Duration of Mechanical Ventilation (Days)		Length of ICU Stay (Days)		Length of Hospital Stay (Days)	
		RR (95% CI)	*p*-Value for Subgroup Differences	MD (95% CI)	*p*-Value for Subgroup Differences	MD (95% CI)	*p*-Value for Subgroup Differences	MD (95% CI)	*p*-Value for Subgroup Differences
**Trial Design**			0.002		0.45		0.58		0.90
	Double-blind	0.80 (0.63, 1.01)		−1.34 (−4.94, 2.25)		−2.83 (−6.49, 0.82)		−2.07 (−4.80, 0.65)	
	Single-blind	0.36 (0.24, 0.53)		0.21 (−0.23, 0.65)		−1.65 (−2.56, −0.74)		Not estimable	
	Open-label	0.74 (0.58, 0.95)		−2.85 (−9.15, 3.45)		3.03 (−8.05, 14.11)		−3.37 (−24.36, 17.62)	
**Type of Intervention**			0.09		0.75		0.73		0.67
	Probiotic	0.77 (0.63, 0.96)		−1.43 (−5.63, 2.78)		−1.73 (−5.25, 1.80)		−0.73 (−2.97, 1.51)	
	Synbiotic	0.50 (0.32, 0.79)		−0.71 (−2.24, 0.82)		−2.45 (−4.50, −0.40)		−2.67 (−11.38, 6.04)	
**Type of comparator**			0.55		0.96		0.47		0.90
	Placebo	0.71 (0.54, 0.94)		−1.12 (−3.41, 1.16)		−2.71 (−5.07, −0.34)		−2.07 (−4.80, 0.65)	
	Others	0.64 (0.49, 0.82)		−1.27 (−6.83, 4.28)		−0.95 (−5.09, 3.19)		−3.37 (−24.36, 17.62)	
**Definition of VAP**			0.20		NA		NA		NA
	Clinically undiagnosed	0.71 (0.49, 1.02)							
	Microbiologically confirmed	0.72 (0.55, 0.94)							
	Inexplicable diagnostic criteria	0.42 (0.24, 0.72)							

VAP, ventilator-associated pneumonia; CI, confidence interval; MD, mean difference; RR, risk ratio; NA, not applicable.

## Data Availability

The data that support the findings of this study are available from the corresponding author, HAC, upon reasonable request.

## Supplementary Materials

The following supporting information can be downloaded at: https://www.mdpi.com/article/10.3390/nu14081600/s1, Table S1: Prisma 2020 checklist; Table S2: Search strategies for online databases; Figure S1: Funnel plot for incidence of VAP; Figure S2: Effect of probiotics on the duration of mechanical ventilation: Figure S3: Funnel plot for the duration of mechanical ventilation; Figure S4: Effect of probiotics on the length of ICU stay: Figure S5: Funnel plot for the length of ICU stay; Figure S6: Effect of probiotics on the length of hospital stay; Figure S7: Doi plot for the length of hospital stay: Figure S8: Effect of probiotics on: (a) ICU mortality; (b) Hospital mortality; (c) 28/30-day mortality; Figure S9: Doi plot for ICU mortality; Figure S10: Doi plot for hospital mortality; Figure S11: Doi plot for 28/30-day mortality; Figure S12: Effect of probiotics on the duration of antibiotic use; Figure S13: Doi plot for the duration of antibiotic use; Figure S14: Effect of probiotics on the incidence of diarrhoea; Figure S15: Doi plot for the incidence of diarrhoea; Figure S16. Sensitivity analysis by excluding studies at some concerns of bias in the randomization process.
