# Supplementary material for: Probiotics for the Prevention of Ventilator-Associated Pneumonia: An Updated Systematic Review and Meta-Analysis of Randomised Controlled Trials
Source: Nutrients. 2022 Apr 12;14(8):1600. doi: 10.3390/nu14081600 (PMC9031795; doi:10.3390/nu14081600)

Supplementary Material

Probiotics for the prevention of ventilator-associated pneumonia: an updated systematic review and meta-analysis of randomized controlled trials

Supplementary Table S1. Prisma 2020 checklist

Supplementary Table S2. Search strategies for online databases

Supplementary Figure S1. Funnel plot for incidence of VAP

Supplementary Figure S2. Effect of probiotics on the duration of mechanical ventilation

Supplementary Figure S3. Funnel plot for the duration of mechanical ventilation

Supplementary Figure S4. Effect of probiotics on the length of ICU stay

Supplementary Figure S5. Funnel plot for the length of ICU stay

Supplementary Figure S6. Effect of probiotics on the length of hospital stay

Supplementary Figure S7. Doi plot for the length of hospital stay

Supplementary Figure S8. Effect of probiotics on: a) ICU mortality; b) Hospital mortality; c) 28/30-day mortality

Supplementary Figure S9. Doi plot for ICU mortality

Supplementary Figure S10. Doi plot for hospital mortality

Supplementary Figure S11. Doi plot for 28/30-day mortality

Supplementary Figure S12. Effect of probiotics on the duration of antibiotic use

Supplementary Figure S13. Doi plot for the duration of antibiotic use

Supplementary Figure S14. Effect of probiotics on the incidence of diarrhoea

Supplementary Figure S15. Doi plot for the incidence of diarrhoea

Supplementary Figure S16. Sensitivity analysis by excluding studies at some concerns of bias in the randomization process

This supplemental material has been provided by the authors to give readers additional information about their work.

Supplementary Table S1. PRISMA 2020 checklist

Abstract

Section and Topic	Item #	Checklist item	Reported (Yes/No)
**TITLE**			
Title	1	Identify the report as a systematic review.	Reported
**BACKGROUND**			
Objectives	2	Provide an explicit statement of the main objective(s) or question(s) the review addresses.	Reported
**METHODS**			
Eligibility criteria	3	Specify the inclusion and exclusion criteria for the review.	Partially reported (250 words limit)
Information sources	4	Specify the information sources (e.g. databases, registers) used to identify studies and the date when each was last searched.	Not reported (250 words limit)
Risk of bias	5	Specify the methods used to assess risk of bias in the included studies.	Not reported (250 words limit)
Synthesis of results	6	Specify the methods used to present and synthesise results.	Not reported (250 words limit)
**RESULTS**			
Included studies	7	Give the total number of included studies and participants and summarise relevant characteristics of studies.	Reported
Synthesis of results	8	Present results for main outcomes, preferably indicating the number of included studies and participants for each. If meta-analysis was done, report the summary estimate and confidence/credible interval. If comparing groups, indicate the direction of the effect (i.e. which group is favoured).	Partially reported (250 words limit)
**DISCUSSION**			
Limitations of evidence	9	Provide a brief summary of the limitations of the evidence included in the review (e.g. study risk of bias, inconsistency and imprecision).	Partially reported (250 words limit)
Interpretation	10	Provide a general interpretation of the results and important implications.	Reported
**OTHER**			
Funding	11	Specify the primary source of funding for the review.	No funding received
Registration	12	Provide the register name and registration number.	Reported

Manuscript

Section and Topic	Item #	Checklist item	Location where item is reported
**TITLE**			
Title	1	Identify the report as a systematic review.	1
**ABSTRACT**			
Abstract	2	See the PRISMA 2020 for Abstracts checklist.	Suppl.
**INTRODUCTION**			
Rationale	3	Describe the rationale for the review in the context of existing knowledge.	3, 4
Objectives	4	Provide an explicit statement of the objective(s) or question(s) the review addresses.	4
**METHODS**			
Eligibility criteria	5	Specify the inclusion and exclusion criteria for the review and how studies were grouped for the syntheses.	5, 6
Information sources	6	Specify all databases, registers, websites, organisations, reference lists and other sources searched or consulted to identify studies. Specify the date when each source was last searched or consulted.	5
Search strategy	7	Present the full search strategies for all databases, registers and websites, including any filters and limits used.	Suppl.
Selection process	8	Specify the methods used to decide whether a study met the inclusion criteria of the review, including how many reviewers screened each record and each report retrieved, whether they worked independently, and if applicable, details of automation tools used in the process.	5, 6
Data collection process	9	Specify the methods used to collect data from reports, including how many reviewers collected data from each report, whether they worked independently, any processes for obtaining or confirming data from study investigators, and if applicable, details of automation tools used in the process.	5, 6
Data items	10a	List and define all outcomes for which data were sought. Specify whether all results that were compatible with each outcome domain in each study were sought (e.g. for all measures, time points, analyses), and if not, the methods used to decide which results to collect.	6, 7
	10b	List and define all other variables for which data were sought (e.g. participant and intervention characteristics, funding sources). Describe any assumptions made about any missing or unclear information.	6, 7
Study risk of bias assessment	11	Specify the methods used to assess risk of bias in the included studies, including details of the tool(s) used, how many reviewers assessed each study and whether they worked independently, and if applicable, details of automation tools used in the process.	7
Effect measures	12	Specify for each outcome the effect measure(s) (e.g. risk ratio, mean difference) used in the synthesis or presentation of results.	7
Synthesis methods	13a	Describe the processes used to decide which studies were eligible for each synthesis (e.g. tabulating the study intervention characteristics and comparing against the planned groups for each synthesis (item #5)).	7
	13b	Describe any methods required to prepare the data for presentation or synthesis, such as handling of missing summary statistics, or data conversions.	7, 8
	13c	Describe any methods used to tabulate or visually display results of individual studies and syntheses.	7, 8
	13d	Describe any methods used to synthesize results and provide a rationale for the choice(s). If meta-analysis was performed, describe the model(s), method(s) to identify the presence and extent of statistical heterogeneity, and software package(s) used.	7, 8
	13e	Describe any methods used to explore possible causes of heterogeneity among study results (e.g. subgroup analysis, meta-regression).	8
	13f	Describe any sensitivity analyses conducted to assess robustness of the synthesized results.	8
Reporting bias assessment	14	Describe any methods used to assess risk of bias due to missing results in a synthesis (arising from reporting biases).	7, 8
Certainty assessment	15	Describe any methods used to assess certainty (or confidence) in the body of evidence for an outcome.	8

Section and Topic	Item #	Checklist item	Location where item is reported
**RESULTS**			
Study selection	16a	Describe the results of the search and selection process, from the number of records identified in the search to the number of studies included in the review, ideally using a flow diagram.	9, Figure 1
	16b	Cite studies that might appear to meet the inclusion criteria, but which were excluded, and explain why they were excluded.	Figure 1
Study characteristics	17	Cite each included study and present its characteristics.	9, Table 1
Risk of bias in studies	18	Present assessments of risk of bias for each included study.	9, Figure 2.
Results of individual studies	19	For all outcomes, present, for each study: (a) summary statistics for each group (where appropriate) and (b) an effect estimate and its precision (e.g. confidence/credible interval), ideally using structured tables or plots.	Figures, suppl.
Results of syntheses	20a	For each synthesis, briefly summarise the characteristics and risk of bias among contributing studies.	9-11
	20b	Present results of all statistical syntheses conducted. If meta-analysis was done, present for each the summary estimate and its precision (e.g. confidence/credible interval) and measures of statistical heterogeneity. If comparing groups, describe the direction of the effect.	9-11
	20c	Present results of all investigations of possible causes of heterogeneity among study results.	12, Table 3
	20d	Present results of all sensitivity analyses conducted to assess the robustness of the synthesized results.	12
Reporting biases	21	Present assessments of risk of bias due to missing results (arising from reporting biases) for each synthesis assessed.	10
Certainty of evidence	22	Present assessments of certainty (or confidence) in the body of evidence for each outcome assessed.	9-11, Table 2
**DISCUSSION**			
Discussion	23a	Provide a general interpretation of the results in the context of other evidence.	13-15
	23b	Discuss any limitations of the evidence included in the review.	15
	23c	Discuss any limitations of the review processes used.	15
	23d	Discuss implications of the results for practice, policy, and future research.	16
**OTHER INFORMATION**			
Registration and protocol	24a	Provide registration information for the review, including register name and registration number, or state that the review was not registered.	5
	24b	Indicate where the review protocol can be accessed, or state that a protocol was not prepared.	5
	24c	Describe and explain any amendments to information provided at registration or in the protocol.	5
Support	25	Describe sources of financial or non-financial support for the review, and the role of the funders or sponsors in the review.	17
Competing interests	26	Declare any competing interests of review authors.	17
Availability of data, code and other materials	27	Report which of the following are publicly available and where they can be found: template data collection forms; data extracted from included studies; data used for all analyses; analytic code; any other materials used in the review.	17

Supplementary Table S2. Search strategies for online databases

Ovid MEDLINE(R) and Epub Ahead of Print, In-Process, In-Data-Review & Other Non-Indexed Citations, Daily and Versions(R) <1946 to January, 2021>

1	exp *Pneumonia/	182098
2	exp Pneumonia, Ventilator-Associated/	3865
3	(pneumon* adj6 (ventilat* or respirator* or nosocomial* or bacteri*)).tw.	32321
4	VAP.tw.	4554
5	(ventilator-associated adj3 pneumonia).tw.	5886
6	1 or 2 or 3 or 4 or 5	204130
7	Probiotics/	20449
8	Synbiotics/	801
9	probiotic*.tw.	28863
10	synbiotic*.tw.	1764
11	exp Lactobacillus/	30849
12	exp Bifidobacterium/	6595
13	lactobacil*.tw,nm.	39585
14	(bifidus or bifidobacter*).tw,nm.	12249
15	streptococc*.tw,nm.	106749
16	actococc*.tw,nm.	3
17	leuconostoc*.tw,nm.	2923
18	pediococc*.tw,nm.	2140
19	(beneficial adj3 bacter*).tw.	2899
20	7 or 8 or 9 or 10 or 11 or 12 or 13 or 14 or 15 or 16 or 17 or 18 or 19	176017
21	randomized controlled trial.pt.	550449
22	controlled clinical trial.pt.	94541
23	randomized.ab.	540719
24	placebo.ab.	223187
25	clinical trials as topic.sh.	198124
26	randomly.ab.	369974
27	trial.ti.	251267
28	21 or 22 or 23 or 24 or 25 or 26 or 271407415	

29 exp animals/ not humans.sh. 4915987

30 28 not 29 1294924

31 6 and 20 and 30 506

Embase 1947-Present, updated daily

1 exp *Pneumonia/ 125285

2 exp ventilator associated pneumonia/ 11868

3 (pneumon* adj6 (ventilat* or respirator* or nosocomial* or bacteri*)).tw. 49079

4 VAP.tw. 7594

5 (ventilator-associated adj3 pneumonia).tw. 9058

6 1 or 2 or 3 or 4 or 5 162341

7 Probiotics/ 42741

8 Synbiotics/ 2204

9 probiotic*.tw. 36802

10 synbiotic*.tw. 2191

11 exp Lactobacillus/ 56260

12 exp Bifidobacterium/ 16498

13 lactobacil*.tw. 47295

14 (bifidus or bifidobacter*).tw. 15659

15 streptococc*.tw. 132959

16 lactococc*.tw. 8217

17 leuconostoc*.tw. 3259

18 pediococc*.tw. 2238

19 (beneficial adj3 bacter*).tw. 3175

20 7 or 8 or 9 or 10 or 11 or 12 or 13 or 14 or 15 or 16 or 17 or 18 or 19 233141

21 Randomized controlled trial/ 686270

22 Controlled clinical trial/ 464824

23 random$.ti,ab. 1737066

24 randomization/ 92449

25 intermethod comparison/ 277199

26 placebo.ti,ab. 337091

27 (compare or compared or comparison).ti. 581704

28 ((evaluated or evaluate or evaluating or assessed or assess) and (compare or compared or comparing or comparison)).ab. 2406236

29 (open adj label).ti,ab. 92304

30 ((double or single or doubly or singly) adj (blind or blinded or blindly)).ti,ab. 255820

31 double blind procedure/ 192148

32 parallel group$1.ti,ab. 28424

33 (crossover or cross over).ti,ab. 114883

34 ((assign$ or match or matched or allocation) adj5 (alternate or group$1 or intervention$1 or patient$1 or subject$1 or participant$1)).ti,ab. 369100

35 (assigned or allocated).ti,ab. 434983

36 (controlled adj7 (study or design or trial)).ti,ab. 396159

37 (volunteer or volunteers).ti,ab. 268503

38 human experiment/ 559965

39 trial.ti. 351518

40 21 or 22 or 23 or 24 or 25 or 26 or 27 or 28 or 29 or 30 or 31 or 32 or 33 or 34 or 35 or 36 or 37 or 38 or 39 5652852

41 (random$ adj sampl$ adj7 (cross section$ or questionnaire$1 or survey$ or database$1)).ti,ab. not (comparative study/ or controlled study/ or randomi?ed controlled.ti,ab. or randomly assigned.ti,ab.) 8876

42 Cross-sectional study/ not (randomized controlled trial/ or controlled clinical study/ or controlled study/ or randomi?ed controlled.ti,ab. or control group$1.ti,ab.) 288579

43 (((case adj control$) and random$) not randomi?ed controlled).ti,ab. 19129

44 (Systematic review not (trial or study)).ti. 191504

45 (nonrandom$ not random$).ti,ab. 17515

46 Random field$.ti,ab. 2612

47 (random cluster adj3 sampl$).ti,ab. 1400

48 (review.ab. and review.pt.) not trial.ti. 940638

49 we searched.ab. and (review.ti. or review.pt.) 39062

50 update review.ab. 118

51 (databases adj4 searched).ab. 46484

52 (rat or rats or mouse or mice or swine or porcine or murine or sheep or lambs or pigs or piglets or rabbit or rabbits or cat or cats or dog or dogs or cattle or bovine or monkey or monkeys or trout or marmoset$1).ti. and animal experiment/ 1128717

53 Animal experiment/ not (human experiment/ or human/) 2372363

54 or/41-53 3831856

55 40 not 54 5022886

56 6 and 20 and 55 1514

CENTRAL via The Cochrane Library

#1 MeSH descriptor: [Pneumonia] explode all trees 5114

#2 MeSH descriptor: [Pneumonia, Ventilator-Associated] explode all trees 350

#3 (pneumon* NEAR/6 (ventilat* or respirator* or nosocomial* or bacteri*)):ti,ab,kw 4952

#4 (VAP):ti,ab,kw 1006

#5 (ventilator-associated NEAR/3 pneumonia):ti,ab,kw (Word variations have been searched) 1670

#6 {OR #1-#5} 8668

#7 MeSH descriptor: [Probiotics] this term only2315

#8 MeSH descriptor: [Synbiotics] this term only 182

#9 (probiotic OR synbiotic):ti,ab,kw (Word variations have been searched) 8306

#10 MeSH descriptor: [Lactobacillus] explode all trees 1723

#11 MeSH descriptor: [Bifidobacterium] explode all trees 794

#12 (lactobacil*):ti,ab,kw 5828

#13 ((bifidus or bifidobacter*)):ti,ab,kw 3398

#14 (streptococc*):ti,ab,kw 6093

#15 (actococc*):ti,ab,kw 0

#16 (leuconostoc*):ti,ab,kw 31

#17 (pediococc*):ti,ab,kw 70

#18 ((beneficial NEAR/3 bacter*)):ti,ab,kw 246

#19 {OR #7-#18} 15992

#20 #6 AND #19 687

Supplementary Figure S1. Funnel plot for incidence of VAP

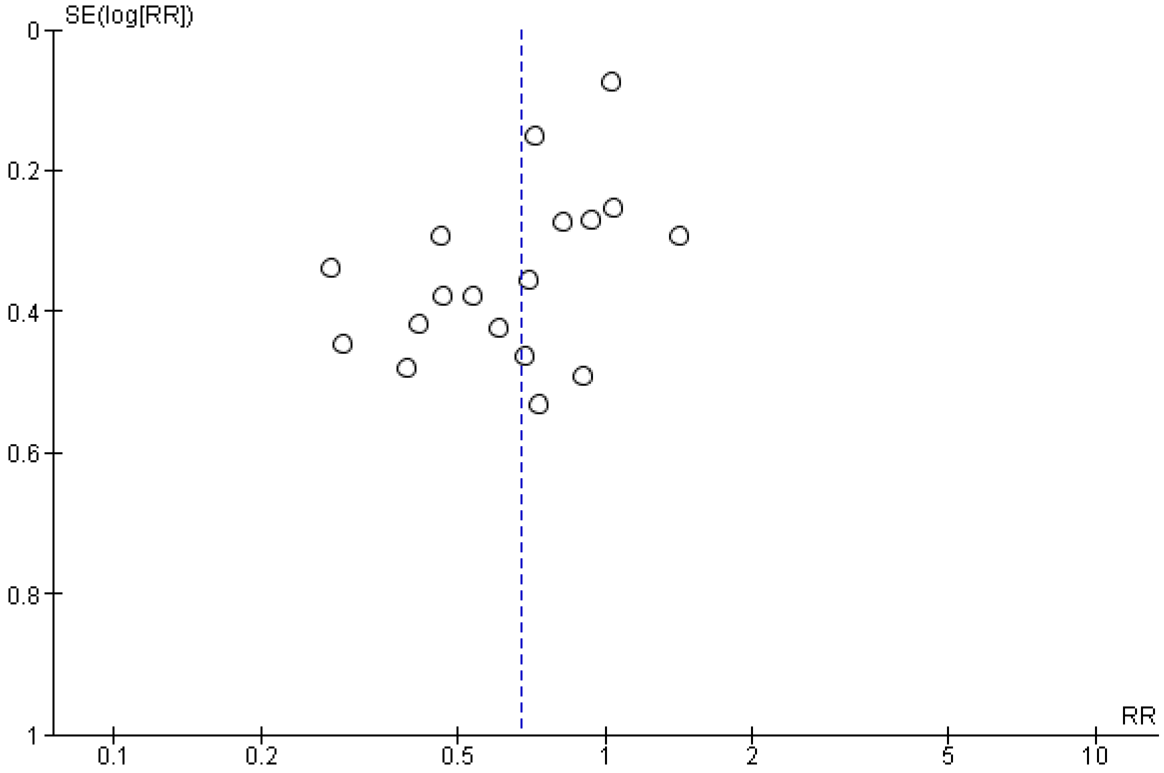

Supplementary Figure S2. Effect of probiotics on the duration of mechanical ventilation

Study or Subgroup	Probiotics Mean	Probiotics SD	Probiotics Total	Control Mean	Control SD	Control Total	Weight	Mean Difference IV, Random, 95% CI
Anandaraj 2019	12.67	3.02	72	7.33	5.29	74	9.8%	5.34 [3.95, 6.73]
Habib 2020	11.6	4.76	32	9.1	3.64	33	9.3%	2.50 [0.44, 4.56]
Johnstone 2021	8	6.67	1318	8	6.67	1312	10.3%	0.00 [-0.51, 0.51]
Klarin 2018	9.9	18.26	69	8.99	16.07	68	5.6%	0.91 [-4.85, 6.67]
Knight 2009	5.33	5.25	130	6.33	6	129	9.9%	-1.00 [-2.37, 0.37]
Mahmoodpoor 2019	8.75	4.79	48	12.08	7.12	54	9.1%	-3.33 [-5.66, -1.00]
Morrow 2010	9.5	6.3	68	9.6	7.2	70	9.2%	-0.10 [-2.36, 2.16]
Nazari 2020	8.19	1.21	73	8	1.51	74	10.3%	0.19 [-0.25, 0.63]
Rongrungruang 2015	39	81.63	75	35.33	73.32	75	0.6%	3.67 [-21.16, 28.50]
Spindler-Vesel 2007	12.2	8.9	26	10.5	7.4	87	7.6%	1.70 [-2.06, 5.46]
Zarinfar 2016	10.3	4.81	30	23.2	0.31	30	9.6%	-12.90 [-14.62, -11.18]
Zeng 2016	13	9.75	118	19.33	11.25	117	8.7%	-6.33 [-9.02, -3.64]
**Total (95% CI)**			**2059**			**2123**	**100.0%**	**-1.22 [-3.25, 0.81]**

Heterogeneity: Tau² = 10.37; Chi² = 308.54, df = 11 (P < 0.00001); I² = 96%
Test for overall effect: Z = 1.18 (P = 0.24)

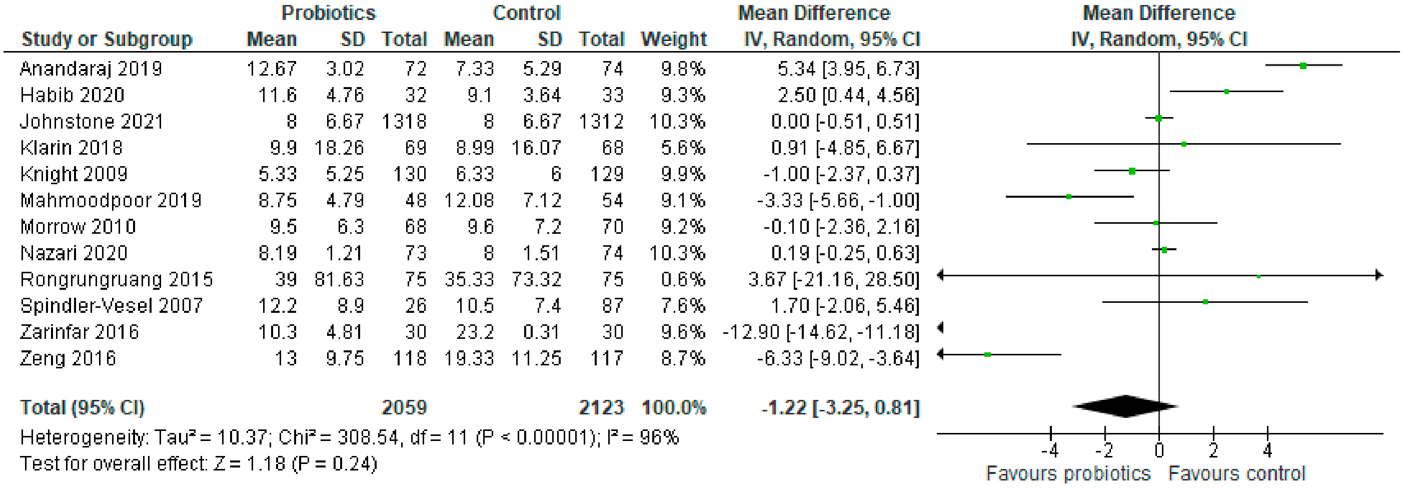

Supplementary Figure S3. Funnel plot for the duration of mechanical ventilation

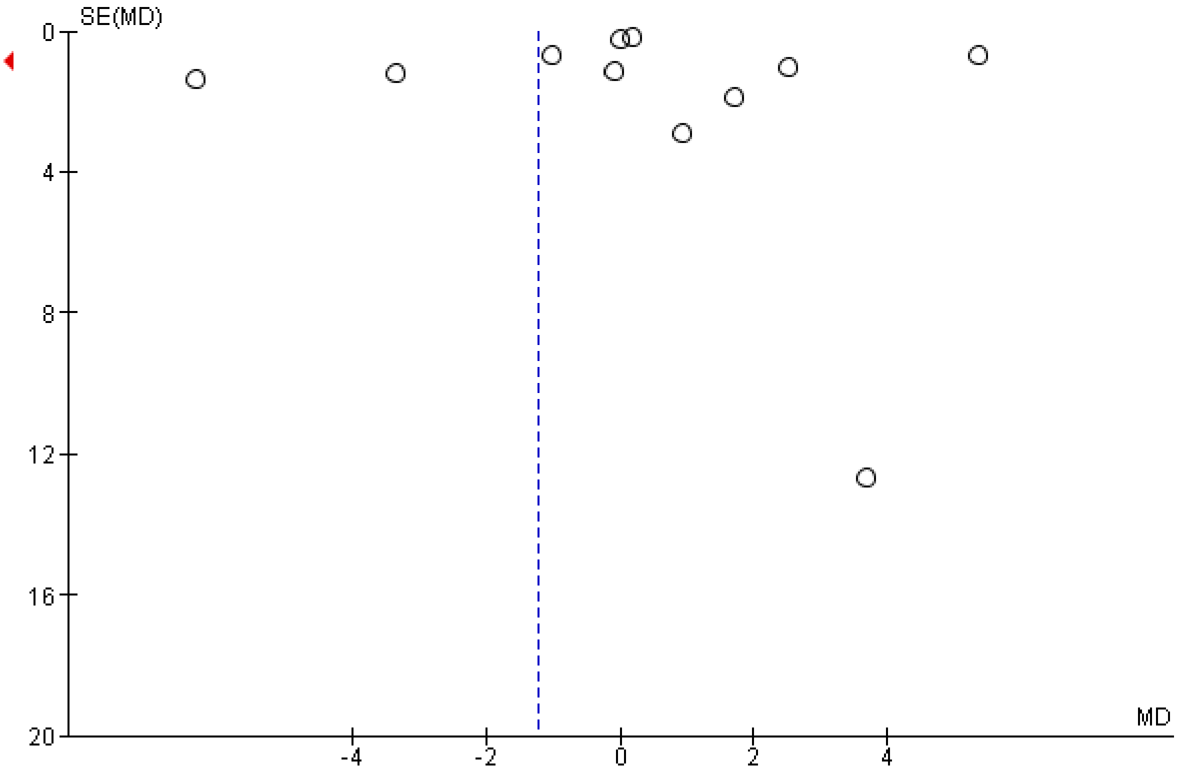

Supplementary Figure S4. Effect of probiotics on the length of ICU stay

Study or Subgroup	Probiotics Mean	Probiotics SD	Probiotics Total	Control Mean	Control SD	Control Total	Weight	Mean Difference IV, Random, 95% CI
Anandaraj 2019	7.17	3.4	72	18.33	5.29	74	8.5%	-11.16 [-12.60, -9.72]
Barraud 2010	18.7	12.4	87	20.2	20.8	80	5.4%	-1.50 [-6.75, 3.75]
Habib 2020	14.6	4.78	32	12.63	3.68	33	8.1%	1.97 [-0.11, 4.05]
Johnstone 2021	12.67	8.89	1318	12.67	7.41	1332	8.9%	0.00 [-0.62, 0.62]
Klarin 2018	7.6	6.02	69	6.55	4.98	68	8.3%	1.05 [-0.80, 2.90]
Knight 2009	6.67	6	130	8	8.24	129	8.3%	-1.33 [-3.09, 0.43]
Mahmoodpoor 2019	11.6	8	48	18.6	6.3	54	7.5%	-7.00 [-9.82, -4.18]
Morrow 2010	14.8	11.8	68	14.6	11.6	70	6.6%	0.20 [-3.71, 4.11]
Nazari 2020	13.35	1.45	73	14.88	1.79	74	8.9%	-1.53 [-2.06, -1.00]
Rongrungruang 2015	44.17	71.05	75	18	18.97	75	1.2%	26.17 [9.53, 42.81]
Shimizu 2018	26.33	15.48	35	30	21.57	37	3.2%	-3.67 [-12.31, 4.97]
Spindler-Vesel 2007	14.1	10	26	13.5	11.5	87	6.0%	0.60 [-3.94, 5.14]
Tan 2011	6.8	3.8	26	10.7	7.3	26	7.2%	-3.90 [-7.06, -0.74]
Zarinfar 2016	14.2	4.7	30	17.6	6.5	30	7.5%	-3.40 [-6.27, -0.53]
Zeng 2016	21.33	13.5	118	29.67	33.76	117	4.4%	-8.34 [-14.92, -1.76]
**Total (95% CI)**			**2207**			**2286**	**100.0%**	**-2.22 [-4.17, -0.28]**

Heterogeneity: Tau² = 11.01; Chi² = 248.54, df = 14 (P < 0.00001); I² = 94%
Test for overall effect: Z = 2.24 (P = 0.03)

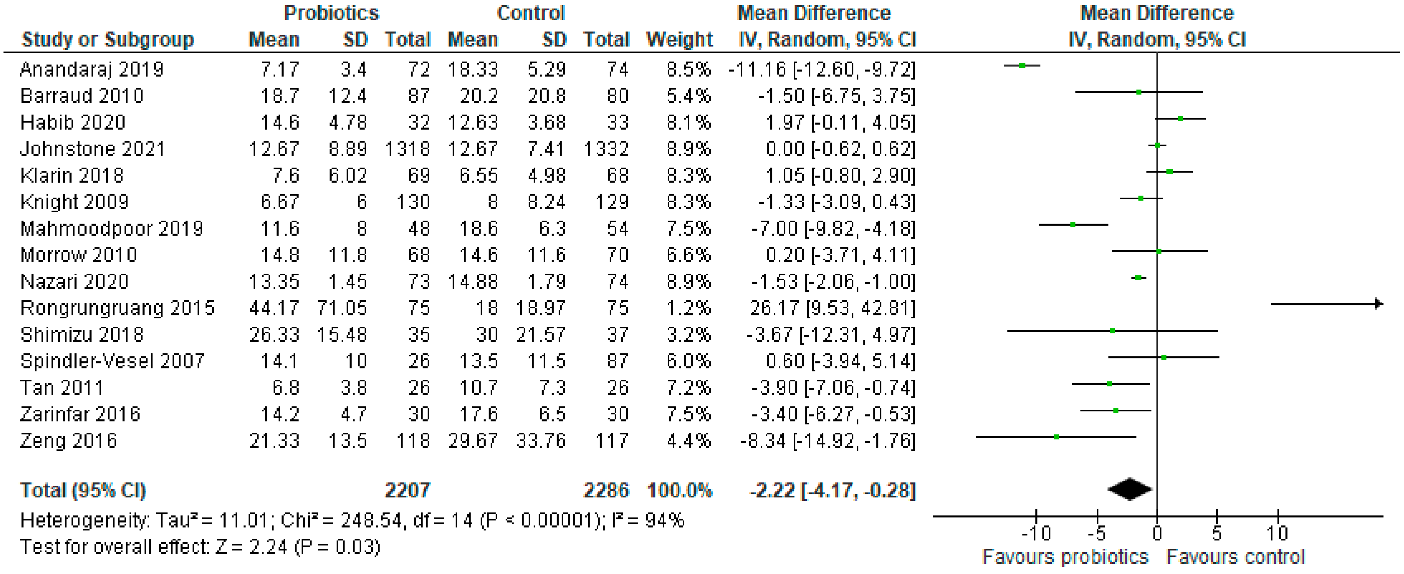

Supplementary Figure S5. Funnel plot for the length of ICU stay

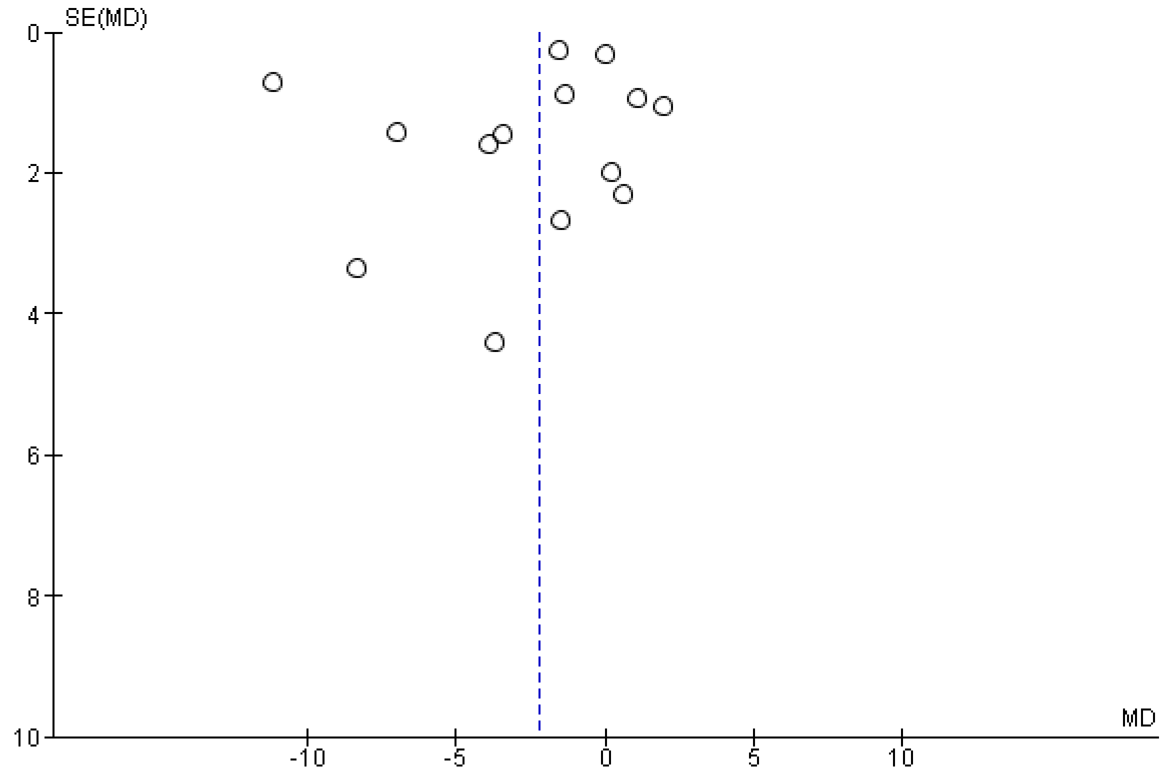

Supplementary Figure S6. Effect of probiotics on the length of hospital stay

	Probiotics			Control				Mean Difference
**Study or Subgroup**	**Mean**	**SD**	**Total**	**Mean**	**SD**	**Total**	**Weight**	**IV, Random, 95% CI**
Anandaraj 2019	12.5	9.07	72	16	13.6	74	12.8%	-3.50 [-7.24, 0.24]
Barraud 2010	26.6	22.3	87	28.9	26.4	80	7.1%	-2.30 [-9.74, 5.14]
Johnstone 2021	25.67	21.48	1318	25	20	1332	16.3%	0.67 [-0.91, 2.25]
Knight 2009	21	20.99	130	19	18.74	129	10.8%	2.00 [-2.85, 6.85]
Mahmoodpoor 2019	14.2	8.6	48	21.1	5.7	54	14.3%	-6.90 [-9.77, -4.03]
Morrow 2010	21.4	14.9	68	21.7	17.4	70	9.9%	-0.30 [-5.70, 5.10]
Rongrungruang 2015	42.67	78.6	75	64.33	126.98	75	0.6%	-21.66 [-55.46, 12.14]
Zarinfar 2016	24.1	5.6	30	27.4	6.6	30	13.9%	-3.30 [-6.40, -0.20]
Zeng 2016	13.5	12.4	118	10.6	10.2	117	14.3%	2.90 [-0.00, 5.80]
**Total (95% CI)**			**1946**			**1961**	**100.0%**	**-1.47 [-4.06, 1.12]**

Heterogeneity: Tau² = 10.03; Chi² = 34.35, df = 8 (P < 0.0001); I² = 77%
Test for overall effect: Z = 1.11 (P = 0.27)

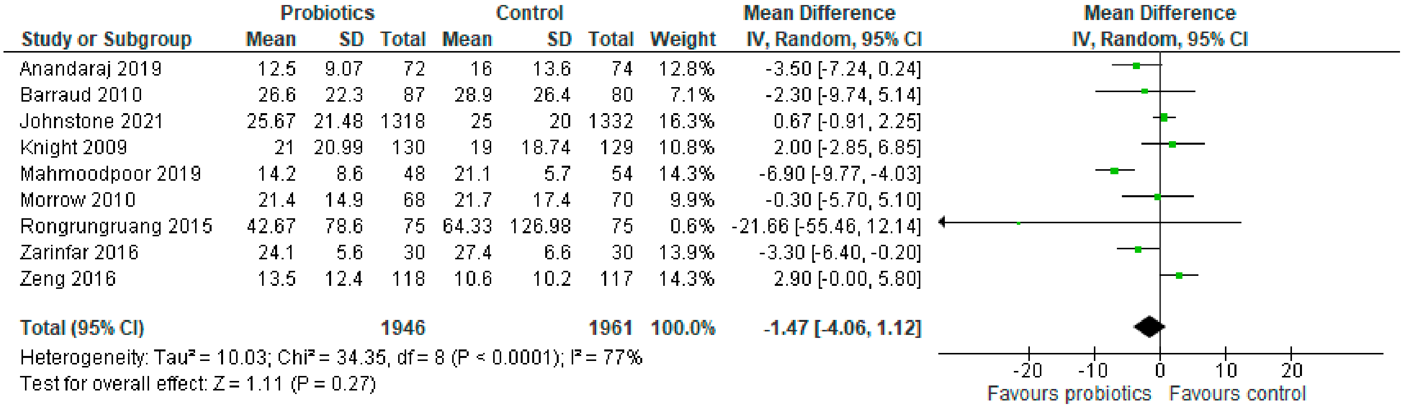

Supplementary Figure S7. Doi plot for the length of hospital stay

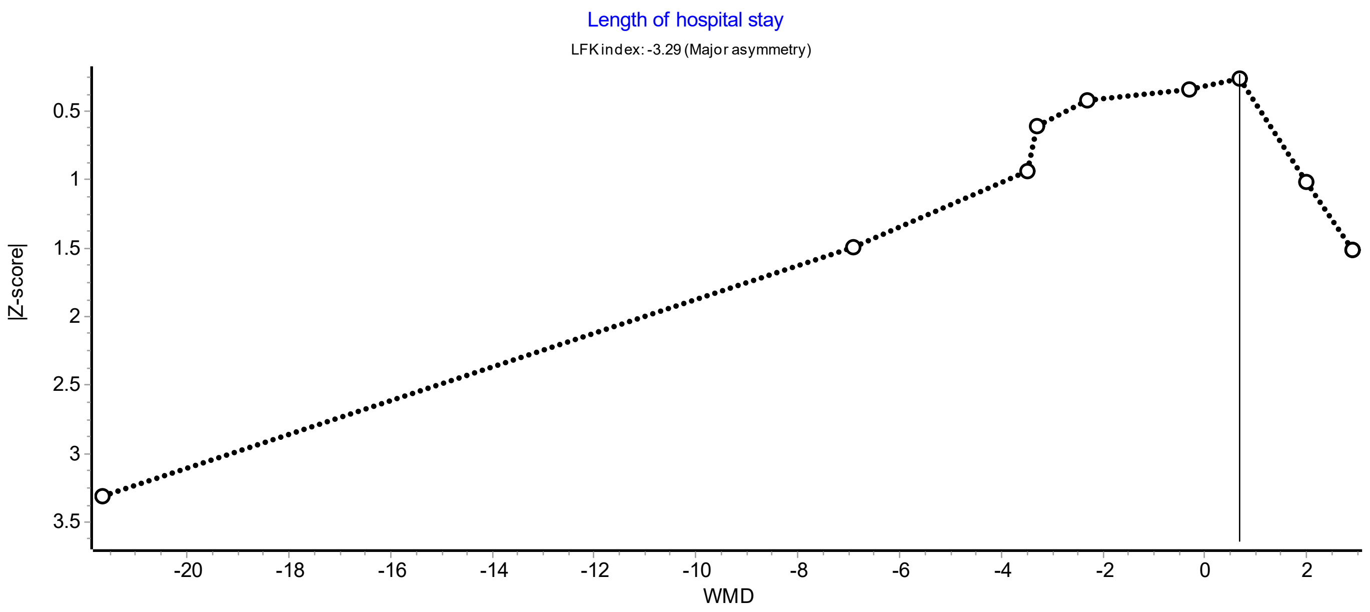

Supplementary Figure S8. Effect of probiotics on: a) ICU mortality; b) Hospital mortality; c) 28/30-day mortality

A

Study or Subgroup	Probiotics Events	Probiotics Total	Control Events	Control Total	Weight	Risk Ratio IV, Random, 95% CI
Anandaraj 2019	22	72	20	74	5.7%	1.13 [0.68, 1.89]
Barraud 2010	21	87	21	80	5.4%	0.92 [0.54, 1.55]
Habib 2020	11	32	12	33	3.4%	0.95 [0.49, 1.83]
Johnstone 2021	279	1318	296	1332	71.0%	0.95 [0.82, 1.10]
Klarin 2018	10	69	11	68	2.4%	0.90 [0.41, 1.97]
Knight 2009	28	130	34	129	7.8%	0.82 [0.53, 1.26]
Mahmoodpoor 2019	5	48	6	52	1.2%	0.90 [0.29, 2.77]
Spindler-Vesel 2007	2	26	5	87	0.6%	1.34 [0.28, 6.50]
Zeng 2016	15	118	9	117	2.4%	1.65 [0.75, 3.63]
**Total (95% CI)**		**1900**		**1972**	**100.0%**	**0.96 [0.85, 1.09]**
Total events	393		414			

Heterogeneity: Tau² = 0.00; Chi² = 3.00, df = 8 (P = 0.93); I² = 0%
Test for overall effect: Z = 0.64 (P = 0.52)

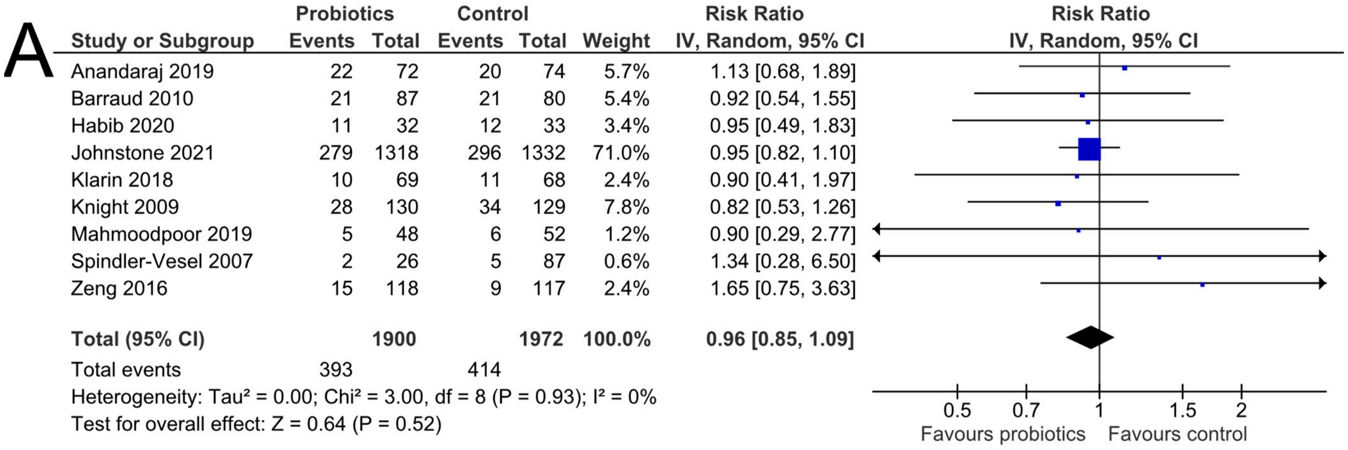

B

Study or Subgroup	Probiotics Events	Probiotics Total	Control Events	Control Total	Weight	Risk Ratio IV, Random, 95% CI
Anandaraj 2019	28	72	30	74	7.1%	0.96 [0.64, 1.43]
Johnstone 2021	363	1318	381	1332	76.7%	0.96 [0.85, 1.09]
Klarin 2018	14	69	12	68	2.4%	1.15 [0.57, 2.30]
Knight 2009	35	130	42	129	8.0%	0.83 [0.57, 1.21]
Morrow 2010	12	68	15	70	2.5%	0.82 [0.42, 1.63]
Shimizu 2018	3	35	4	37	0.6%	0.79 [0.19, 3.29]
Zarinfar 2016	2	30	6	30	0.5%	0.33 [0.07, 1.52]
Zeng 2016	11	103	16	108	2.2%	0.72 [0.35, 1.48]
**Total (95% CI)**		**1825**		**1848**	**100.0%**	**0.94 [0.84, 1.05]**
Total events	468		506			

Heterogeneity: Tau² = 0.00; Chi² = 3.44, df = 7 (P = 0.84); I² = 0%
Test for overall effect: Z = 1.15 (P = 0.25)

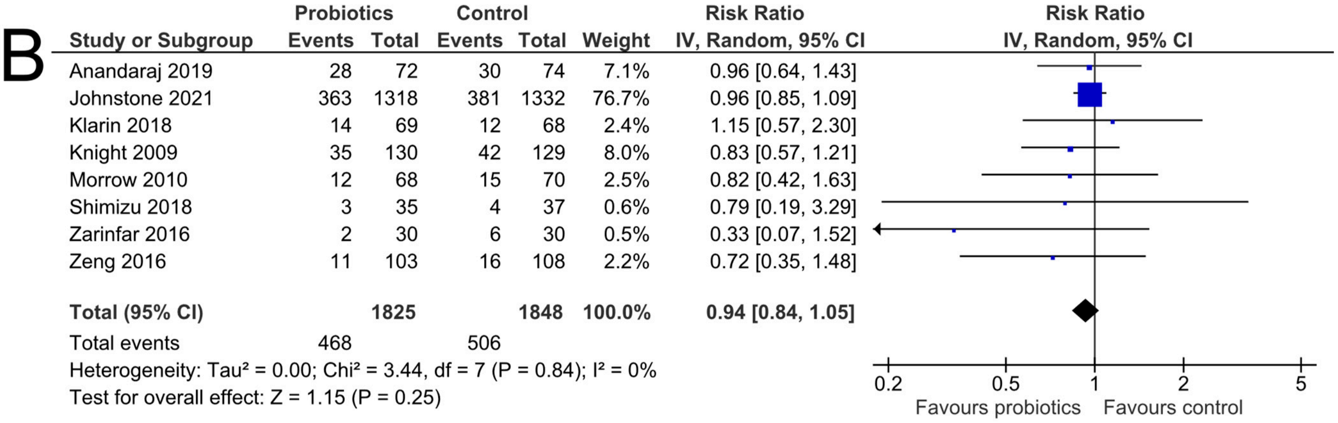

C

Study or Subgroup	Probiotics Events	Probiotics Total	Control Events	Control Total	Weight	Risk Ratio IV, Random, 95% CI
Barraud 2010	22	87	19	80	41.6%	1.06 [0.62, 1.82]
Giamarellos-Bourboulis 2009	5	36	10	36	12.6%	0.50 [0.19, 1.32]
Rongrungruang 2015	18	75	17	75	35.2%	1.06 [0.59, 1.89]
Tan 2011	3	26	5	26	6.8%	0.60 [0.16, 2.26]
Tsilika 2021	3	59	2	53	3.9%	1.35 [0.23, 7.76]
**Total (95% CI)**		**283**		**270**	**100.0%**	**0.94 [0.66, 1.32]**
Total events	51		53			

Heterogeneity: Tau² = 0.00; Chi² = 2.61, df = 4 (P = 0.63); I² = 0%
Test for overall effect: Z = 0.37 (P = 0.71)

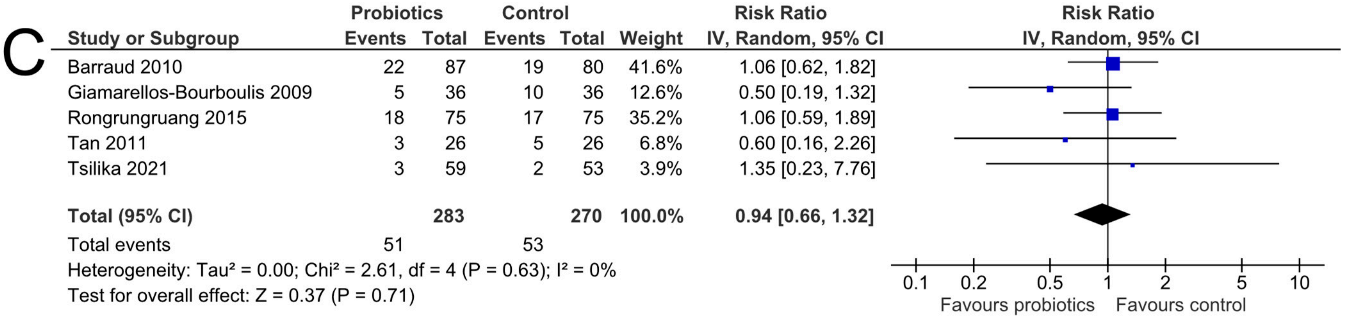

Supplementary Figure S9. Doi plot for ICU mortality

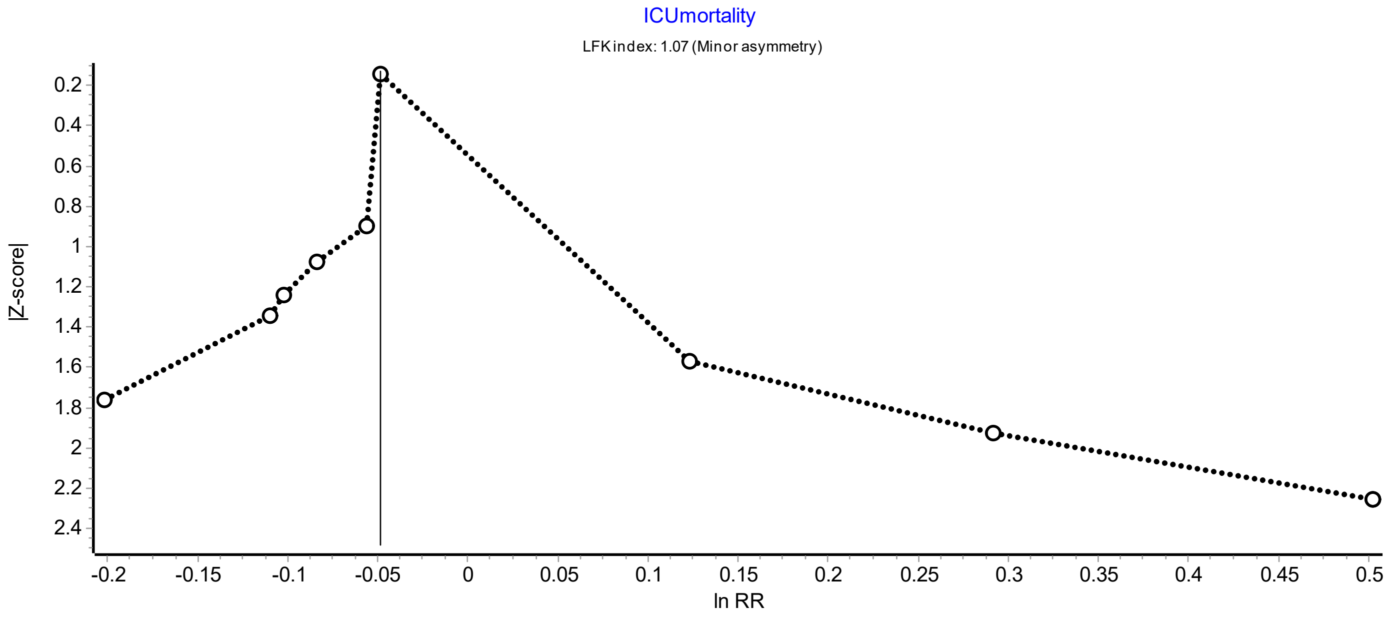

Supplementary Figure S10. Doi plot for hospital mortality

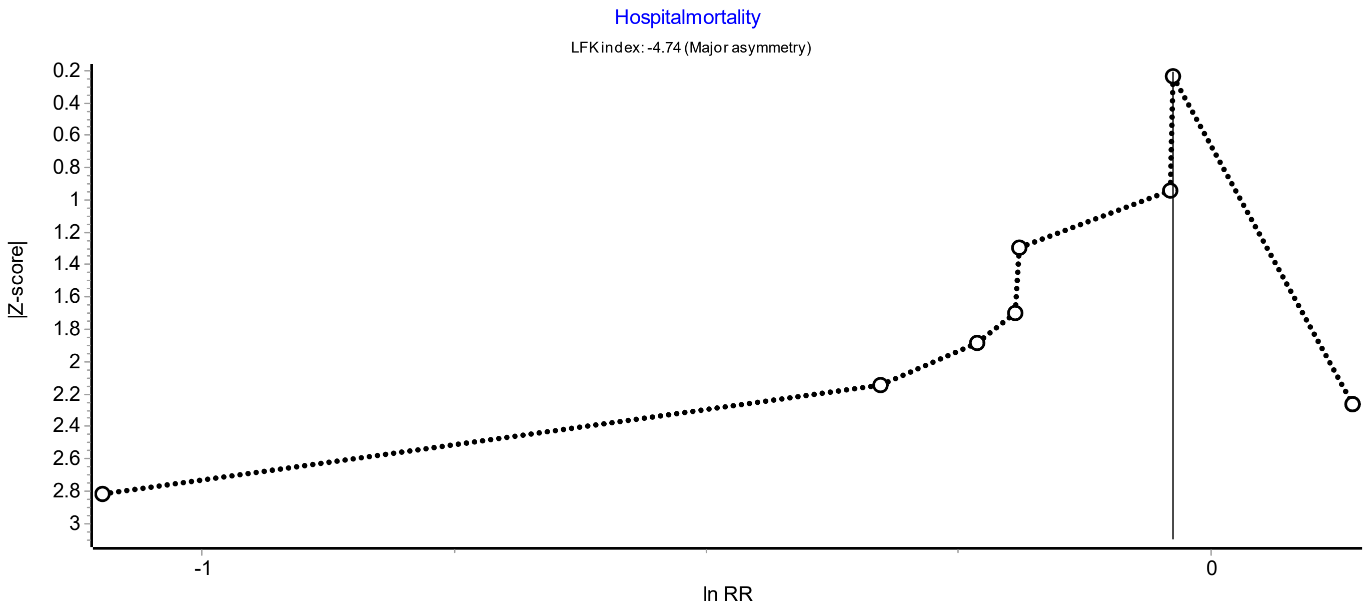

Supplementary Figure S11. Doi plot for 28/30-day mortality

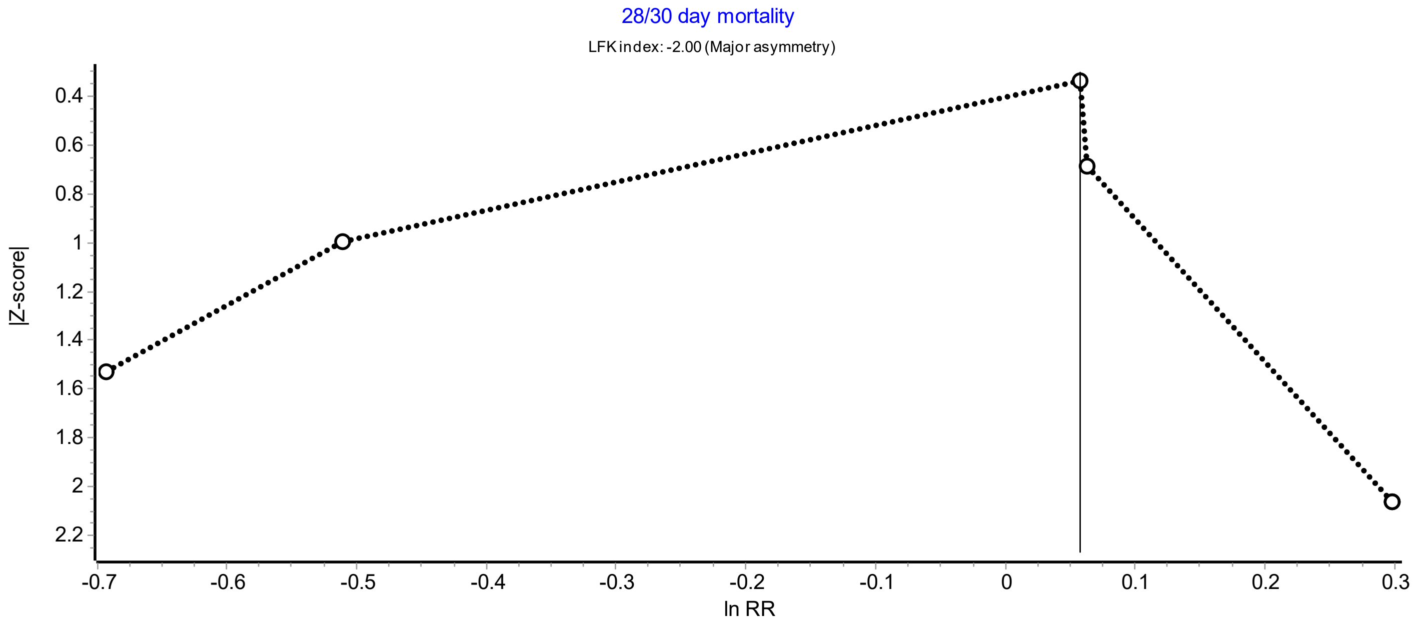

Supplementary Figure S12. Effect of probiotics on the duration of antibiotic use

	Probiotics			Control				Mean Difference
Study or Subgroup	Mean	SD	Total	Mean	SD	Total	Weight	IV, Random, 95% CI
Morrow 2010	13.3	10.4	68	16.3	14.4	70	2.2%	-3.00 [-7.18, 1.18]
Shimizu 2018	15	12.38	35	18.67	13.87	37	1.0%	-3.67 [-9.74, 2.40]
Tan 2011	11.9	4.9	26	14.1	6	26	4.2%	-2.20 [-5.18, 0.78]
Zeng 2016	6.8	2	118	7.94	2.9	117	92.6%	-1.14 [-1.78, -0.50]
**Total (95% CI)**			**247**			**250**	**100.0%**	**-1.25 [-1.86, -0.64]**

Heterogeneity: Tau² = 0.00; Chi² = 1.79, df = 3 (P = 0.62); I² = 0%
Test for overall effect: Z = 4.00 (P < 0.0001)

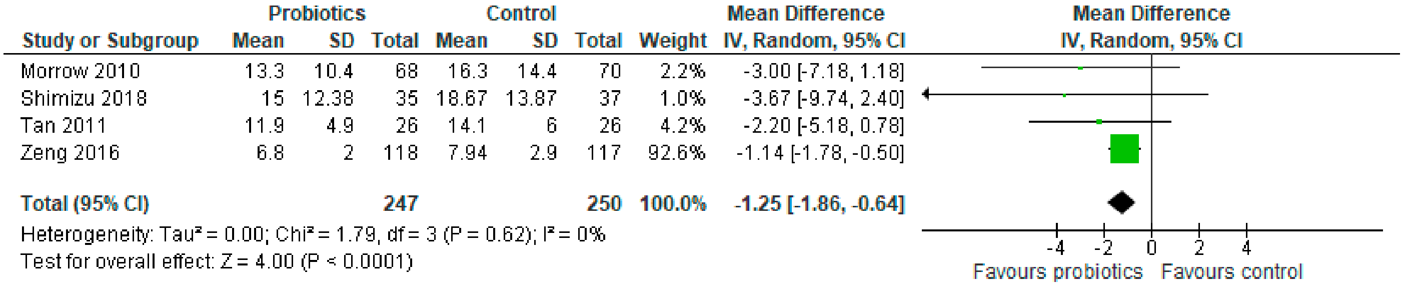

Supplementary Figure S13. Doi plot for the duration of antibiotic use

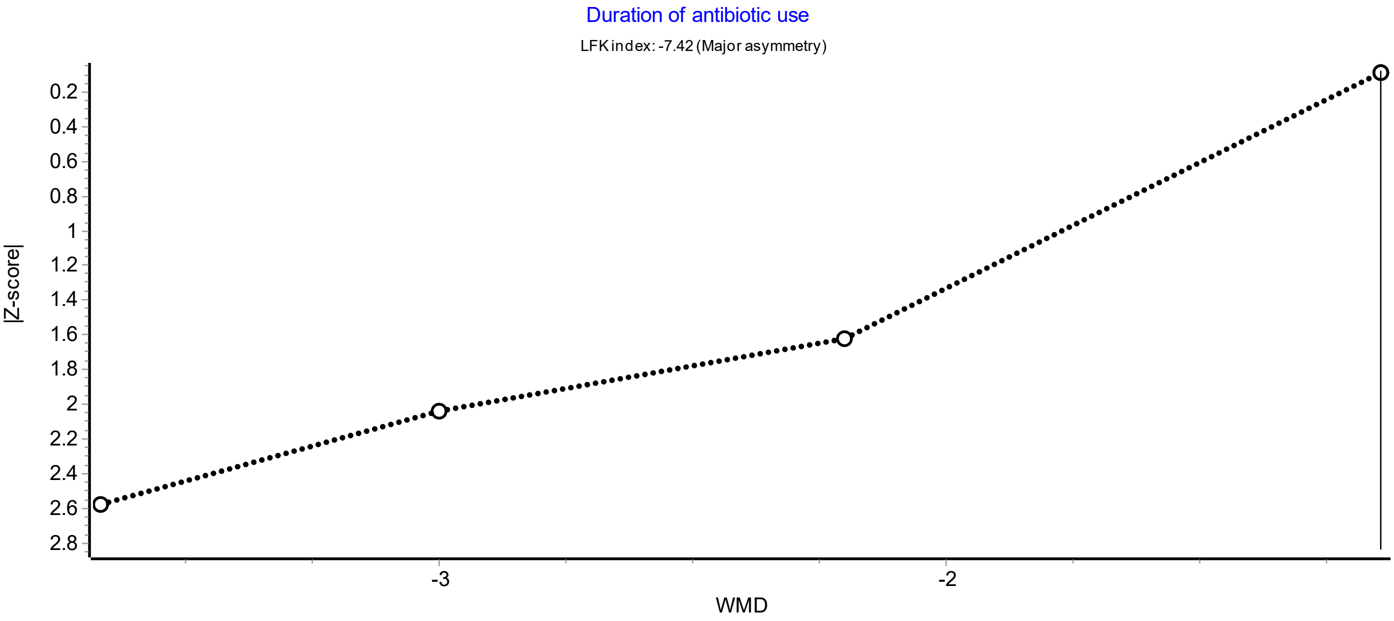

Supplementary Figure S14. Effect of probiotics on the incidence of diarrhoea

Study or Subgroup	Probiotics Events	Probiotics Total	Control Events	Control Total	Weight	Risk Ratio IV, Random, 95% CI
Barraud 2010	48	87	42	80	15.1%	1.05 [0.79, 1.39]
Johnstone 2021	785	1318	787	1332	52.6%	1.01 [0.95, 1.07]
Knight 2009	7	130	9	129	1.7%	0.77 [0.30, 2.01]
Mahmoodpoor 2019	7	48	15	54	2.3%	0.53 [0.23, 1.18]
Morrow 2010	42	68	44	70	17.0%	0.98 [0.76, 1.27]
Rongrungruang 2015	19	75	14	75	4.0%	1.36 [0.74, 2.50]
Shimizu 2018	2	35	10	37	0.8%	0.21 [0.05, 0.90]
Tsilika 2021	0	59	2	53	0.2%	0.18 [0.01, 3.67]
Zarinfar 2016	15	30	17	30	6.3%	0.88 [0.55, 1.42]
**Total (95% CI)**		**1850**		**1860**	**100.0%**	**0.98 [0.86, 1.11]**
Total events	925		940			

Heterogeneity: Tau² = 0.01; Chi² = 9.82, df = 8 (P = 0.28); I² = 19%
Test for overall effect: Z = 0.33 (P = 0.74)

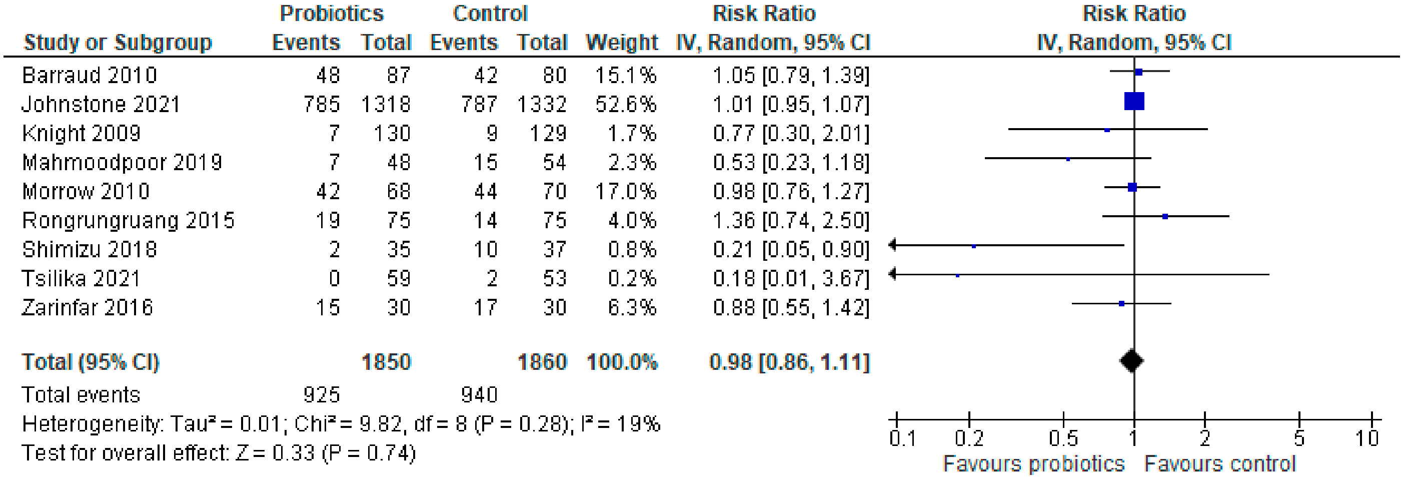

Supplementary Figure S15. Doi plot for the incidence of diarrhoea

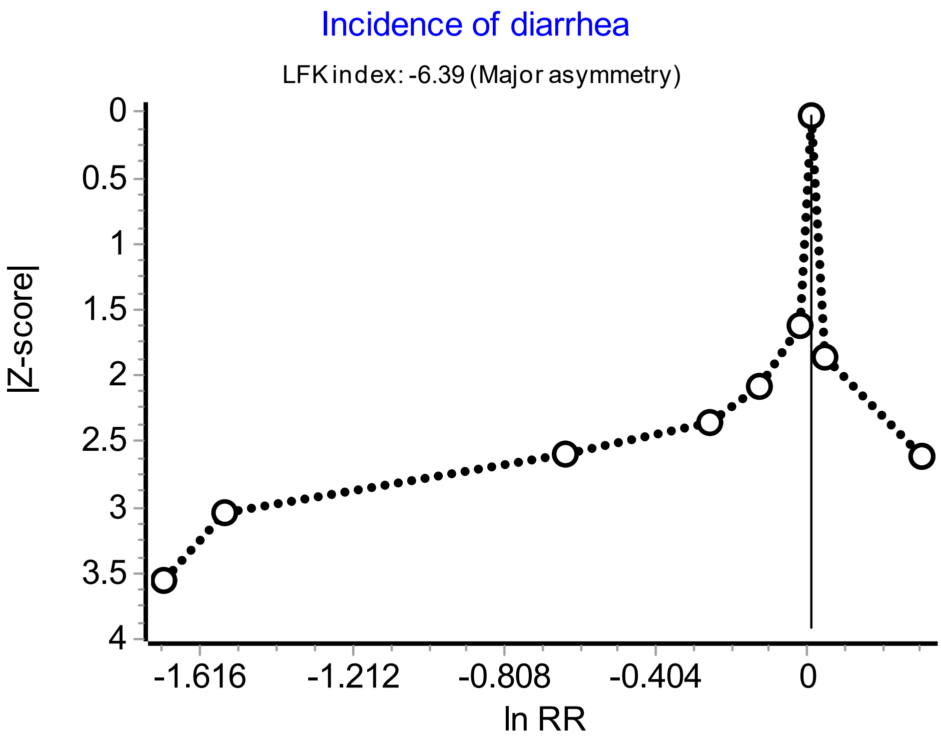

Supplementary Figure S16. Sensitivity analysis by excluding studies at some concerns of bias in the randomization process

Study or Subgroup	Probiotics Events	Probiotics Total	Control Events	Control Total	Weight	Risk Ratio IV, Random, 95% CI
Anandaraj 2019	7	72	8	74	5.4%	0.90 [0.34, 2.35]
Barraud 2010	23	87	15	80	10.4%	1.41 [0.79, 2.51]
Forestier 2008	24	102	24	106	12.0%	1.04 [0.63, 1.71]
Giamarellos-Bourboulis 2009	15	36	16	36	11.3%	0.94 [0.55, 1.60]
Habib 2020	5	32	7	33		Not estimable
Johnstone 2021	289	1318	284	1332	20.2%	1.03 [0.89, 1.19]
Klarin 2018	7	69	10	68	5.9%	0.69 [0.28, 1.71]
Knight 2009	12	130	17	129	8.4%	0.70 [0.35, 1.41]
Mahmoodpoor 2019	7	48	13	54		Not estimable
Morrow 2010	13	73	28	73		Not estimable
Nazari 2020	9	73	33	74		Not estimable
Rongrungruang 2015	18	75	22	75		Not estimable
Shimizu 2018	5	35	18	37	6.2%	0.29 [0.12, 0.71]
Spindler-Vesel 2007	4	26	34	87	5.6%	0.39 [0.15, 1.01]
Tan 2011	7	26	13	26	7.8%	0.54 [0.26, 1.13]
Tsilika 2021	7	59	15	53	6.8%	0.42 [0.19, 0.95]
Zarinfar 2016	7	30	15	30		Not estimable
Zeng 2016	43	118	59	117		Not estimable
**Total (95% CI)**		**1960**		**2028**	**100.0%**	**0.78 [0.60, 1.00]**
Total events	400		454			

Heterogeneity: Tau² = 0.08; Chi² = 20.68, df = 10 (P = 0.02); I² = 52%
Test for overall effect: Z = 1.93 (P = 0.05)

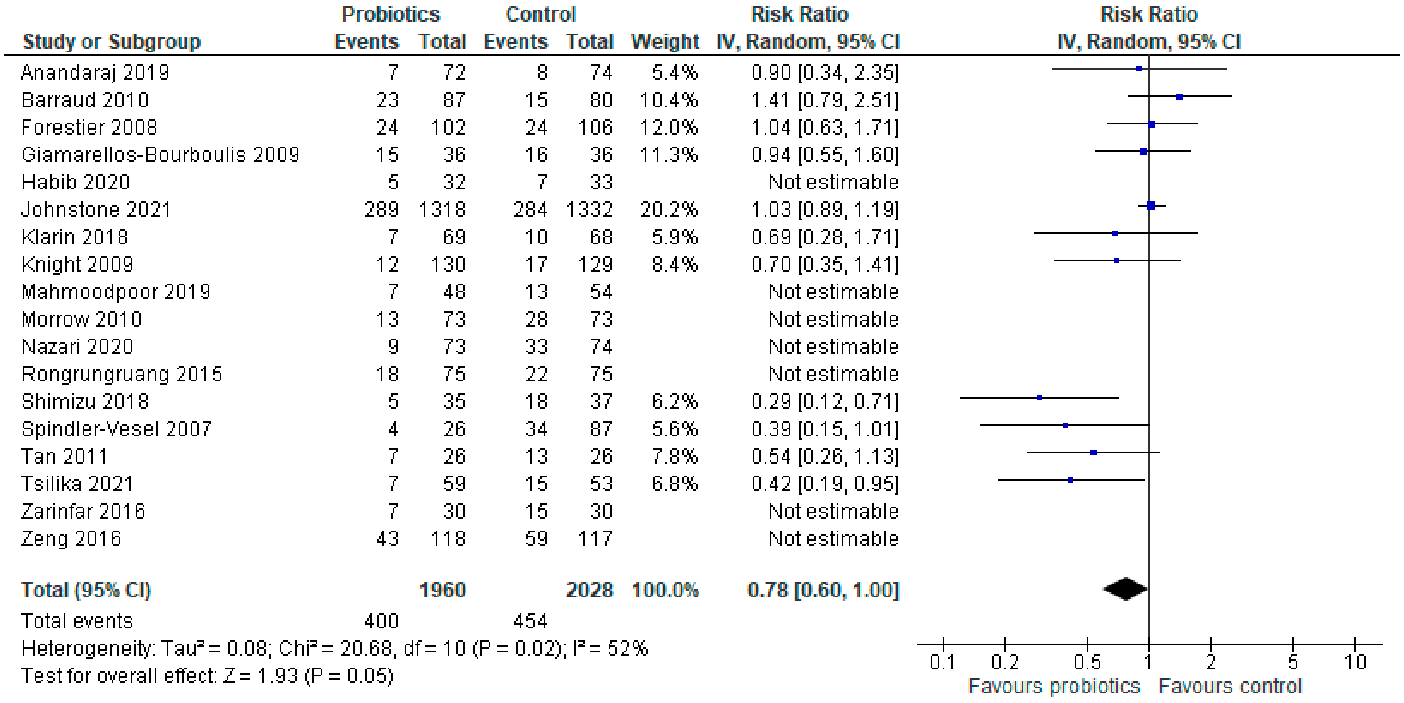

Supplement: Supplementary file 1 [file nutrients-14-01600-s001.zip › nutrients-1670561-supplementary.pdf]
